# Comprehensive transcriptomic profiling reveals tissue-specific molecular signatures and dysregulated pathways in human diabetic foot ulcers

**DOI:** 10.3389/fendo.2025.1669205

**Published:** 2025-11-03

**Authors:** Guangrong Hu, Ayesha Nisar, Sawar Khan, Wen Li, Enfang Zhu, Haoling Cui, Guiqin Zhang, Yonghan He, Hui Sun

**Affiliations:** ^1^ Department of Emergency, The Second Affiliated Hospital of Harbin Medical University, Harbin, Heilongjiang, China; ^2^ State Key Laboratory of Genetic Evolution & Animal Models, Key Laboratory of Healthy Aging Research of Yunnan Province, Kunming Institute of Zoology, Chinese Academy of Sciences, Kunming, Yunnan, China; ^3^ Department of Cell Biology, School of Life Sciences, Central South University, Changsha, Hunan, China; ^4^ Institute of Molecular Biology and Biotechnology, The University of Lahore, Lahore, Pakistan; ^5^ Department of Endocrinology, The Second Affiliated Hospital of Dali University (The Third People’s Hospital of Yunnan Province), Kunming, Yunnan, China

**Keywords:** diabetic foot ulcer, diabetic associated complications, transcriptomics of diabetic foot ulcer, pathways of diabetic foot ulcer, pathogenesis of diabetic foot ulcer

## Abstract

**Background:**

Diabetic foot ulcers (DFUs) are a severe complication of diabetes mellitus characterized by impaired wound healing, chronic inflammation, and tissue degeneration. We sought to identify tissue specific molecular drivers of DFU pathogenesis across skin, adipose, and muscle compartments.

**Methods:**

High throughput RNA sequencing was performed on skin, adipose, and muscle tissues from DFU patients and non-ulcerated diabetic controls. Differential expression analyses and pathway enrichment were conducted to delineate common and compartment-specific transcriptional changes.

**Results:**

All DFU tissues exhibited a conserved upregulation of immune activation genes—including chemokines (*CXCL1-8*), cytokines (*IL1B, IL6*), and NF-κB pathway components—alongside downregulation of metabolic regulators (*PPARG, ADIPOQ*), oxidative phosphorylation genes (*SDHA, NDUFS2*), and insulin signaling factors (*IRS1, AKT2*). Skin showed increased keratinocyte proliferation and senescence markers (*KRT16, FOXM1*); adipose tissue revealed adipocyte dedifferentiation and elevated matrix protease activity (*MMP9*); and muscle displayed fibrotic remodeling and mitochondrial suppression (*COL1A1, NDUFS7*). Enrichment analyses implicated IL17 signaling, PPAR pathways, and cellular senescence as central disrupted processes.

**Conclusion:**

DFUs are driven by a dual pathology of inflammatory amplification and metabolic shutdown, overlaid with distinct tissue-specific alterations. Key targets such as chemokine signaling, PPAR-mediated metabolism, and senescence factors emerge as promising candidates for precision therapies aimed at restoring inflammatory–metabolic balance and enhancing wound healing.

## Graphical Abstract

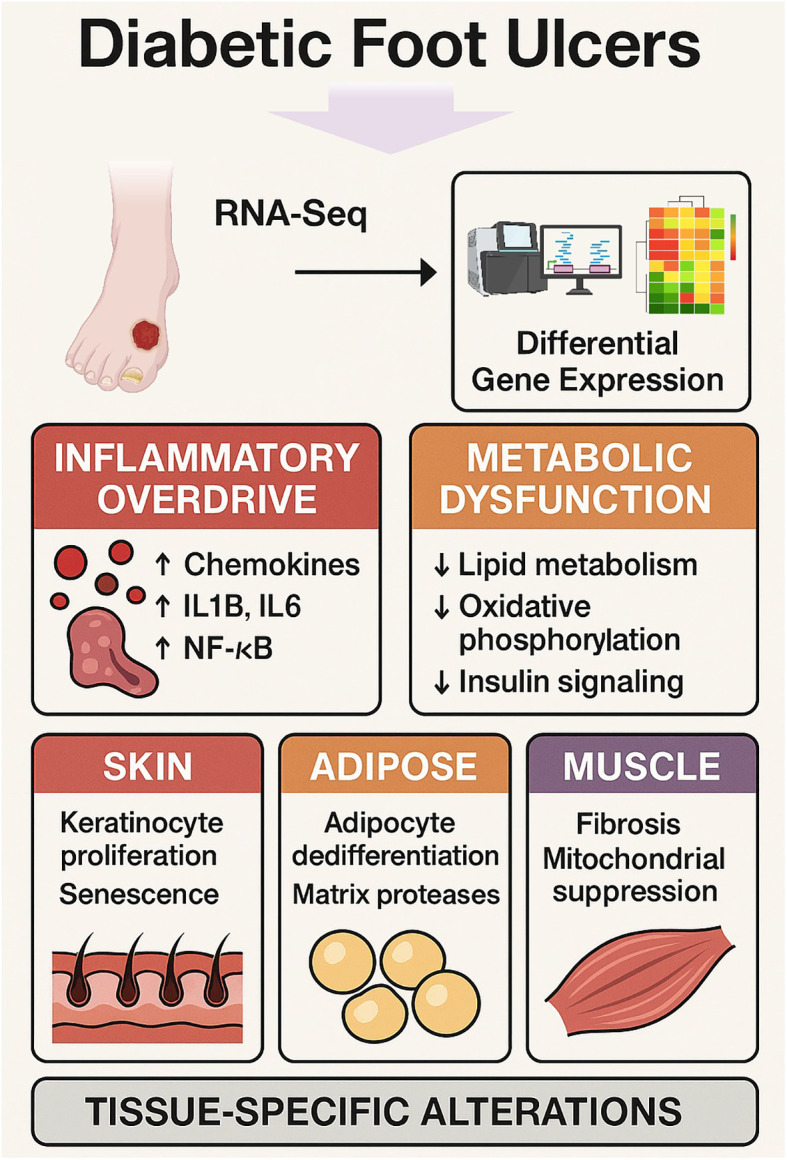

## 1 Introduction

Diabetic foot ulcers (DFUs) are among the most debilitating complications of diabetes mellitus (DM), affecting approximately 15%–25% of diabetic patients during their lifetime (1). DFUs represent chronic, non-healing wounds typically resulting from a combination of peripheral neuropathy, impaired immune response, vascular insufficiency, and metabolic disturbances (2). These ulcers pose a significant health burden due to their high recurrence rate, prolonged healing time, and potential progression to severe complications, including amputations and systemic infections, dramatically reducing quality of life and increasing healthcare costs globally (3). Despite substantial advancements in clinical management, current therapeutic interventions are often limited by incomplete understanding of the complex and multifactorial molecular pathology underlying DFU formation and persistence (4).

At a cellular level, DFUs are characterized by chronic inflammation, dysregulated tissue remodeling, impaired angiogenesis, and sustained cellular senescence (5, 6). Chronic inflammatory processes, primarily mediated by cytokines, chemokines, and growth factors, contribute significantly to delayed wound healing and persistent tissue injury. Elevated pro-inflammatory cytokines, including interleukin-6 (*IL6*), tumor necrosis factor-alpha (*TNF-α*), and various chemokines such as *CXCL8* (*IL8*), have been consistently reported in DFU tissues, suggesting their central roles in perpetuating chronic inflammation (7–9). Moreover, recent studies indicate that cellular senescence, marked by stable cell-cycle arrest and the secretion of pro-inflammatory mediators (the senescence-associated secretory phenotype, SASP), significantly exacerbates the inflammatory environment, impairing wound repair mechanisms (10–12). Therefore, elucidating detailed inflammatory and senescent molecular signatures within specific tissue layers remains essential to develop targeted therapeutic approaches.

In addition to inflammatory and senescent processes, impaired lipid metabolism and mitochondrial dysfunction are emerging as pivotal contributors to DFU pathogenesis (13–16). Metabolic disturbances associated with diabetes include disrupted lipid handling and alterations in adipose tissue function, leading to abnormal fat deposition and chronic low-grade inflammation (17, 18). Central to these metabolic processes is the peroxisome proliferator-activated receptor gamma (PPARG) signaling pathway, a critical regulator of lipid metabolism, adipogenesis, insulin sensitivity, and inflammation. Dysfunctional PPARG signaling and associated pathways have been implicated in exacerbating metabolic derangements and inflammatory responses, further impairing tissue regeneration (19, 20). Consequently, exploring lipid metabolic dysfunctions and their impact on DFU progression, particularly within adipose and muscle tissues, could significantly enhance our understanding of DFU pathology (21, 22).

Furthermore, diabetic neuropathy profoundly influences DFU formation and chronicity, characterized by impaired sensory perception, diminished pain response, and compromised neural regulation of vascular and immune functions (23, 24). Neuroactive ligand-receptor interactions regulate neural communication, modulate inflammation, and influence vascular dynamics essential for normal wound healing (25). Dysfunction in neuroactive signaling pathways may thus critically impact the pathological progression of DFUs, yet comprehensive molecular evidence detailing such dysregulations remains sparse. Investigating these neural regulatory impairments through integrative transcriptomics could clarify their role in the persistent and complex nature of DFUs.

Given the multifactorial and heterogeneous nature of DFUs, a comprehensive molecular profiling approach across different tissue layers (skin, fat, and muscle) could reveal distinct yet interconnected pathological processes underlying chronic diabetic wounds. Recent advances in RNA sequencing (RNA-seq) technology and sophisticated bioinformatics pipelines offer unprecedented opportunities to systematically dissect tissue-specific transcriptomic signatures (26, 27). Such analyses facilitate the identification of critical regulatory genes, functional pathways, and molecular interactions, significantly enhancing the mechanistic understanding of DFU pathology and guiding the development of innovative therapeutic strategies.

This study aimed to comprehensively characterize transcriptomic alterations across skin, adipose, and muscle tissues derived from diabetic foot ulcer patients compared to matched non-ulcerated diabetic controls. Through rigorous RNA-seq analysis, we systematically identified differentially expressed genes (DEGs), conducted detailed functional enrichment analyses, and mapped critical molecular pathways involved in DFU pathogenesis. By examining inflammatory, senescence-associated, metabolic, and neural regulatory pathways in a tissue-specific context, we sought to elucidate shared and unique molecular dysregulations underlying DFU pathogenesis. This integrative analysis aimed not only to expand current understanding but also to identify novel molecular targets potentially exploitable for therapeutic interventions aimed at mitigating DFU progression, promoting effective wound repair, and ultimately improving clinical outcomes for diabetic patients. Our comprehensive analysis revealed critical insights into tissue-specific molecular disruptions, including pronounced inflammatory responses driven by cytokine and chemokine pathways, robust senescence-associated molecular signatures, significant metabolic impairments in lipid and energy metabolism, and substantial alterations in neuroactive ligand-receptor interactions. This transcriptomic landscape underscores the complexity of DFU pathology and highlights interconnected pathogenic mechanisms warranting further exploration.

## 2 Methods

### 2.1 Study design and sample collection

A total of 52 subjects were recruited for this study, including 26 patients diagnosed with diabetic foot ulcers (DFU) and 26 control subjects (CTRL). The ethical approval for this study was obtained from the Ethical Review Board at the Third People’s Hospital of Yunnan Province (approval number: 2023KY046), and all procedures conformed to the principles outlined in the Declaration of Helsinki. Written informed consent was obtained from each participant before sample collection.

Tissue samples of the experimental group were collected below the knee joints, specifically targeting skin, fat, and muscle tissues surrounding active wounds. For the CTRL group, tissue samples were obtained from patients undergoing surgical treatment for various orthopedic conditions. Biopsies were collected from the following sites: right/left femoral neck fracture, right tibiofibular fracture after surgery, left femur fracture after surgery, right lateral epidermal osteoma and osteoarthritis, comminuted fracture of the proximal left ulna, bilateral femoral head ischemic necrosis, severe osteoarthritis of the left knee, developmental dysplasia of the right hip with total dislocation, left femoral head ischemic necrosis, right femoral head necrosis, and comminuted fracture. Before tissue extraction, rigorous clinical preparation involving disinfection, deiodination, and debridement was performed, preserving the complete structural integrity of each wound. Tissue specimens measuring approximately 0.5 cm² each were surgically excised from defined regions adjacent to the wounds. Collected tissues were immediately placed into labeled, sterile, transparent centrifuge tubes, separately containing skin, adipose, and muscle tissues. All samples were snap-frozen and stored at −80 °C until subsequent RNA extraction and analysis.

#### 2.1.1 Inclusion/exclusion criteria of diseased and control participants

Participants in the experimental group were adults aged 18–70 years diagnosed with type 2 diabetes mellitus for at least two years, with foot ulcers classified as Wagner grade ≥3 located below both knees. Patients with conditions or factors that could affect blood glucose levels (e.g., Cushing’s syndrome, recent surgery, or medications), diabetes diagnosed during pregnancy or lactation, organ failure, malignant tumors, or specific infections were excluded.

The CTRL group consisted of age-matched adults within the same 18–70-year range whose lower limbs required surgical treatment but who had normal glucose levels (random blood glucose <11.1 mmol/L, fasting blood glucose <6.1 mmol/L, or HbA1c <6.5%). Controls were excluded if they had conditions potentially causing persistent hyperglycemia, gouty arthritis with glucocorticoid use, acute or chronic osteomyelitis, septic arthritis, bone tumors, congenital bone deformities, organ failure, malignant tumors, or specific infections.

### 2.2 RNA extraction and library preparation

Total RNA was extracted from the samples using TRIzol reagent (Invitrogen, Thermo Fisher Scientific), following the manufacturer’s protocol. RNA quality and integrity were evaluated using an Agilent 2100 Bioanalyzer (Agilent Technologies), and only samples with RNA integrity numbers (RIN) above 7 were selected for library preparation. Poly(A)-enriched RNA sequencing (RNA-seq) libraries were prepared using standard Illumina protocols involving poly(A)-mRNA isolation, fragmentation, first and second-strand cDNA synthesis, adaptor ligation, and PCR amplification.

Sequencing was performed on an Illumina NovaSeq X Plus platform to generate paired-end reads of 150 bp. Raw sequencing data were subjected to quality control analyses, and adaptor sequences were removed using trim_galore (version 0.6.7). High-quality clean reads obtained after trimming were used for subsequent bioinformatics analyses.

### 2.3 RNA-seq data processing and alignment

Clean reads obtained after trimming were aligned to the human reference genome (GRCh38) using the STAR aligner (28). Default parameters were employed with minor adjustments to optimize the alignment quality. SAM files generated by STAR were converted into sorted and indexed BAM files using SAMtools (version 1.18; https://github.com/samtools).

Gene expression quantification was performed by counting uniquely mapped reads to annotated gene regions with featureCounts (version 2.0.6; https://subread.sourceforge.net/featureCounts.html). Read counts for each gene were used as the input for subsequent differential expression analyses.

### 2.4 Gene expression data normalization and transformation

Raw gene counts were preprocessed and normalized following the integrated Differential Expression and Pathway (iDEP) analysis pipeline. Initial preprocessing involved filtering out low-expression genes based on the criteria of at least 0.5 counts per million (CPM) in a minimum of one sample. Gene identifiers were standardized using the gene ID conversion functionality of the iDEP pipeline (29, 30).

Normalization of gene expression data was carried out using the counts per million (CPM) method implemented by the EdgeR package (31). Subsequently, normalized counts were transformed using a balanced log transformation to reduce heteroscedasticity and improve the data’s suitability for downstream analysis. Specifically, gene counts were log-transformed using the formula log2(CPM+c), with the constant (c=4) recommended by the iDEP server. The effectiveness of normalization and transformation was assessed by visual inspection of distribution plots, including box plots and density plots, ensuring comparable distributions across samples within each tissue type.

### 2.5 Differential expression analysis and clustering

Differentially expressed genes (DEGs) between DFU and CTRL tissues within each specific tissue type (skin, fat, and muscle) were identified using the DESeq2 R package (32). DESeq2 employs a negative binomial generalized linear model to estimate variance-mean dependence in count data, providing robust statistical inference. Genes were classified as significantly differentially expressed based on adjusted p-values of <0.05 and fold-change criteria of ≥2 (absolute log2 fold change ≥1).

Hierarchical clustering analyses of DEGs were performed using the heatmap.2 function within the R environment to visualize differential expression patterns and sample grouping clearly. Volcano plots depicting the distribution of significant DEGs according to their statistical significance (-log_10_ adjusted p-value) and expression fold-change (log2) were generated using the ggplot2 R package. Genomic distribution plots of DEGs across chromosomes were also generated to illustrate genome-wide alterations in gene expression profiles. Certain genes with high expression were validated through qPCR. The relative mRNA expression levels were calculated using the 2^-ΔΔCt^ method, with GAPDH used as the internal control.

### 2.6 Functional enrichment and pathway analysis

To identify significantly enriched functional categories and biological pathways associated with DEGs, Gene Ontology (GO) and Kyoto Encyclopedia of Genes and Genomes (KEGG) pathway enrichment analyses were conducted using ShinyGO (33). DEGs identified from each tissue were analyzed separately to elucidate tissue-specific functional implications. Statistical significance for enriched terms was determined using false discovery rate (FDR)-corrected p-values, with a significance threshold of FDR ≤0.05.

Furthermore, significantly enriched KEGG pathways identified from DEGs were visualized, and gene mapping was performed to illustrate the involvement of specific differentially regulated genes within each pathway. Genes significantly enriched in each KEGG pathway were annotated clearly to distinguish upregulated (red) and downregulated (green) genes, facilitating the identification of key regulatory genes implicated in DFU pathogenesis.

### 2.7 Western blot analysis

Proteins were extracted from human skin, adipose, and muscle tissue specimens by rinsing with phosphate-buffered saline (PBS) and homogenizing in ice-cold RIPA buffer (BP115DG, Boston BioProducts, Ashland, MA, USA) supplemented with EDTA and EGTA using a tissue homogenizer (KZ-III-FP, Servicebio, Wuhan, China). Homogenates were kept on ice for 30 min and centrifuged at 13,000 rpm for 20 min at 4 °C to remove debris. Protein concentrations in the supernatants were determined with a bicinchoninic acid (BCA) protein assay kit (P0010, Beyotime, Shanghai, China). Equal amounts of protein were mixed with 4× Laemmli sample buffer (1610747, Bio-Rad, Hercules, CA, USA) containing 5% β-mercaptoethanol and denatured at 95 °C for 5 min. Proteins were then separated on 10% SDS-PAGE gels and transferred to 0.22-μm polyvinylidene fluoride (PVDF) membranes (Millipore, Bedford, MA, USA) using a Bio-Rad wet transfer system. Membranes were blocked with 5% (w/v) skim milk prepared in Tris-buffered saline containing 0.1% Tween-20 (TBS-T) for 2 h at room temperature, followed by incubation with primary antibodies Col1a1 (HY-P81227, MedChemExpress, China), MMP9 (24317T, Cell Signaling Technology, China), PPARγ (YP-mAb-03325, Uping Bio, China), and IL17 (YP-mAb-12431, Uping Bio, China) overnight at 4 °C. After several washes, membranes were exposed to species-appropriate horseradish peroxidase (HRP)-conjugated secondary antibodies for 2 h at room temperature. Bands were visualized with the GeneGnome XRQ-NPC System (Synoptics, Frederick, MD, USA) or SCG-W3000 Plus (Servicebio, Wuhan, China) and quantified using ImageJ software (NIH, Bethesda, MD, USA).

### 2.8 Tissue composition and inter-tissue signaling

Computational deconvolution of bulk RNA-seq profiles was performed to estimate the relative abundance of major cell types in skin, adipose, and muscle samples. Transcripts per million (TPM)–normalized data were input into CIBERSORTx (34) using a custom reference matrix derived from publicly available single-cell RNA-seq atlases of human skin, adipose, and skeletal muscle. Default parameters were applied with 100 permutations for significance testing, and cell types with deconvolution p <0.05 were considered reliably estimated. In parallel, cellular enrichment scores were computed using xCell (35) with the xCell64 signature gene set to provide complementary estimates of tissue-resolved cellular composition.

To infer paracrine ligand–receptor interactions across tissue compartments, we employed a two-step strategy. First, lists of differentially upregulated genes were generated for each tissue using our established differential expression pipeline (DESeq2; adjusted p <0.05, log2FC >1). Second, these gene lists were queried against curated ligand–receptor databases CellChat (36) and CellPhoneDB (37) to identify candidate interaction pairs. To prioritize biologically relevant regulatory links, we further applied NicheNet (38), which computes ligand–target regulatory potential scores. For cross-tissue inference, we considered ligands upregulated in one tissue paired with corresponding receptors either upregulated or highly expressed in a second tissue. Only ligand–receptor pairs supported by both databases or those with high NicheNet regulatory scores were retained for downstream analysis. Predicted inter-tissue interactions were visualized using chord diagrams and heatmaps to highlight signaling directionality, functional categories, and tissue-specific paracrine communication patterns.

## 3 Results

### 3.1 Quality control and exploratory analysis of normalized RNA-seq data from diabetic foot ulcers across different tissue types

To assess tissue-specific molecular signatures, we collected skin, fat, and muscle samples from patients diagnosed with DFU. For comparison, the same tissues were obtained from age-matched patients with lower limb fractures, serving as the nDFU control group. The demographic and clinical characteristics of both groups are summarized in **Table 1**. Quality control and exploratory analyses were conducted on RNA-seq data from DFU samples and CRTL tissues derived from skin, fat, and muscle (**Supplementary Figures S1–S3**). RNA-seq data were preprocessed by filtering low-expression genes, normalization, and log2 transformation. The boxplots and density plots revealed that the distributions of transformed gene expression values were comparable across the DFU and CTRL groups in skin (**Supplementary Figures S1A, B**), fat (**Supplementary Figures S2A, B**), and muscle tissues (**Supplementary Figures S3A, B**), indicating effective normalization. Furthermore, sequencing reads predominantly mapped to protein-coding genes across all tissue types, with smaller fractions mapping to immunoglobulin (IG), long non-coding RNA (lncRNA), and mitochondrial rRNA (Mt_rRNA) genes (**Supplementary Figure S1C, S2C, S3C**). Principal component analysis (PCA) plots showed clear clustering of samples by condition in each tissue type, with principal component 1 (PC1) accounting for a substantial proportion of the variance: 39% in skin (**Supplementary Figure S1D**), 45.5% in fat (**Supplementary Figure S2D**), and notably, 73.2% in muscle tissue (**Supplementary Figure S3D**). These distinct clusters reflect substantial differences in transcriptomic profiles between DFU and control samples within each tissue type, especially pronounced in muscle samples. Overall, the results from quality control analyses indicate that the data preprocessing steps were successfully applied, ensuring robust subsequent differential gene expression and enrichment analyses.

**Table 1:** Baseline clinical and biochemical characteristics of patients with diabetic foot ulcer (DFU) and CTRL.

Variables	DFU	nDFU/CTRL	P-value
Gender (M/F)	8/4	8/5	0.88
Age (years)	60.66 ± 11.85	52 ± 10.48	0.064
Duration of diabetes (years)	12.08 ± 6.09	——	——
Body mass index(kg/m^2^)	24.15 ± 3.71	23.49 ± 2.53	0.616
Hemoglobin A1c (%)	8.38 ± 1.38	——	——
Blood glucose (mmol/L)	9.23 ± 3.74	4.66 ± 0.79	0.001
WBC count (10^9/L)	9.86 ± 4.00	7.51 ± 2.15	0.118
Neutrophil count (10^9/L)	7.17 ± 3.96	4.84 ± 1.74	0.136
Lymphocyte count (10^9/L)	1.86 ± 0.82	1.97 ± 0.52	0.965
Neutrophil rate (%)	70.09 ± 11.52	63.3 ± 7.20	0.285
Lymphocyte rate (%)	21.07 ± 9.90	27.54 ± 7.85	0.224
Ultrasensitive C-reactive protein (mg/L)	70.79 ± 59.20	10.53 ± 10.20	0.003
Hemoglobin (g/L)	107.91 ± 24.21	138.58 ± 17.69	0.001
RBC count (10^12/L)	3.78 ± 0.82	4.51 ± 0.45	0.011
Platelet count (10^9/L)	363.33 ± 135.64	269.83 ± 74.53	0.03
Aspartate aminotransferase (U/L)	25.46 ± 34.13	18.48 ± 8.74	0.475
Alanine aminotransferase (U/L)	36.35 ± 68.03	16.47 ± 8.20	0.301
Albumin (g/L)	32.11 ± 5.70	41.71 ± 4.97	0.0001
Urea nitrogen (mmol/L)	8.22 ± 4.64	4.72 ± 1.46	0.031
Serum creatinine (umol/L)	104.33 ± 63.68	72.16 ± 11.77	0.109
Serum uric acid (umol/L)	363.25 ± 112.56	322.58 ± 105.92	0.352
Low density lipoprotein (mmol/L)	2.69 ± 1.18	2.29 ± 0.76	0.403
Total cholesterol (mmol/L)	4.33 ± 1.35	4.26 ± 0.68	0.888
Triglyceride (mmol/L)	1.75 ± 0.58	1.57 ± 0.92	0.414
High density lipoprotein (mmol/L)	0.93 ± 0.40	1.33 ± 0.31	0.007

Data are presented as mean ± standard deviation (SD) for continuous variables and as counts for categorical variables. P-values were calculated to compare differences between groups.

### 3.2 Transcriptomic landscape and functional enrichment analysis of diabetic foot ulcer skin tissue

**Figure 1:**
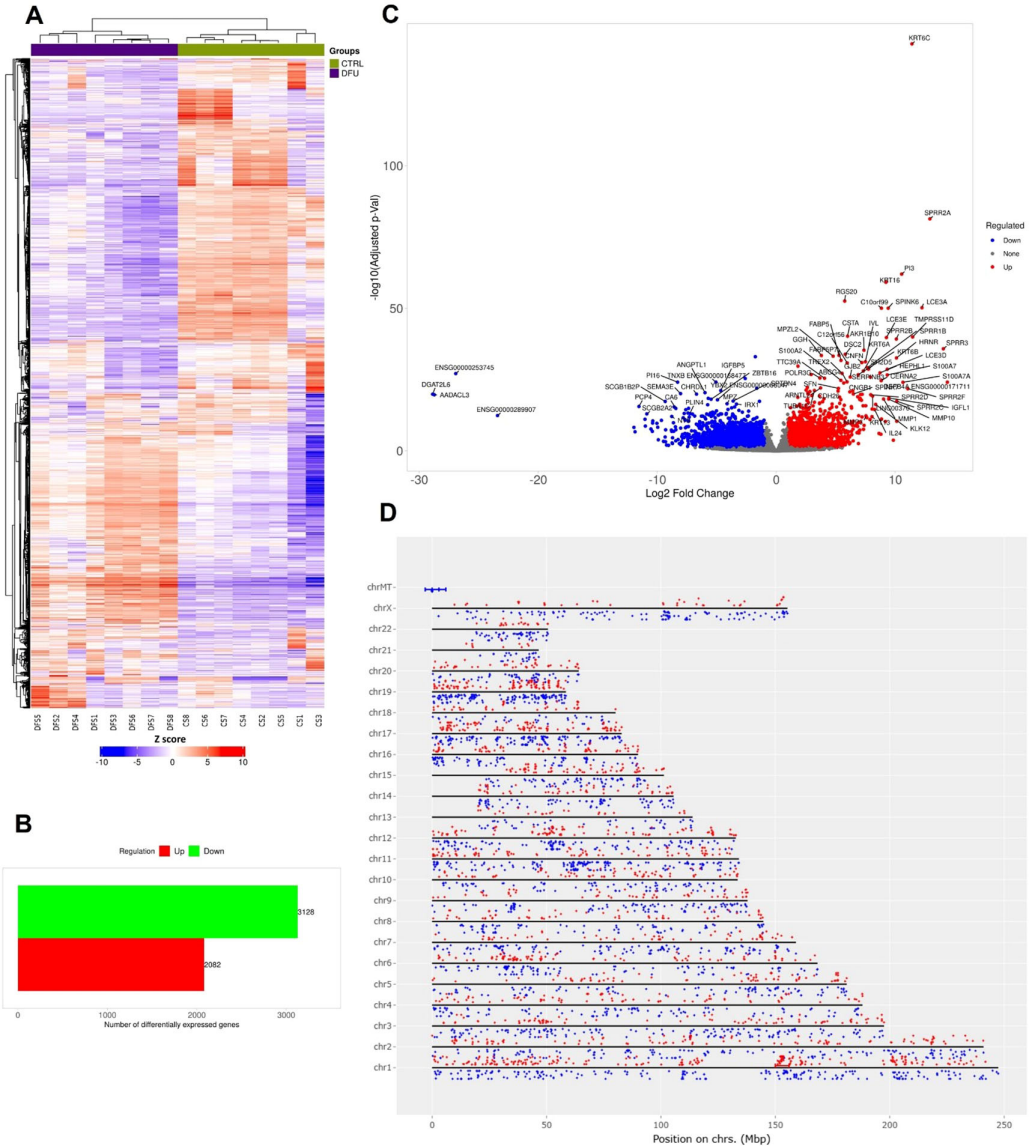
Transcriptomic landscape of diabetic foot ulcer in skin tissue. **(A)** Hierarchical clustering of transcript expression data showing differential gene expression patterns across the DFU and CTRL skin samples. **(B)** Bar graph depicting the summary of differentially expressed genes (DEGs) in the DFU-vs.-CTRL comparison. **(C)** Volcano plot displaying DEGs between DFU and CTRL samples. The x-axis shows log2 fold-change (FC), and the y-axis shows -log10 (adjusted p value). Red dots represent significantly upregulated genes, blue dots represent significantly downregulated genes, and gray dots represent non-significant changes. Top genes with high FC and significance are labeled. **(D)** Genomic distribution of DEGs mapped across human chromosomes. Red dots represent upregulated genes, and blue dots represent downregulated genes. The distribution of dots around the chromosome corresponds the log2 FC values.

The transcriptomic profile of DFU skin displayed marked divergence from that of CTRL tissue. Hierarchical clustering of global gene expression profiles revealed two sharply distinct branches that aligned perfectly with the clinical classifications, highlighting the presence of strong, disease-specific transcriptional signatures (**Figure 1A**). Differential expression analysis identified 5,210 transcripts that met rigorous statistical thresholds (log2 FC >1, FDR <0.05). Notably, the majority of these genes were downregulated in DFU (n = 3,128), while 2,082 were upregulated, suggesting that transcriptional suppression is the dominant regulatory trend in ulcerated skin (**Figure 1B**; **Supplementary Table S1**). The range of deregulation was broad, spanning nearly three orders of magnitude, as visualized in the volcano plot (**Figure 1C**). Highly induced genes included stress-responsive epidermal markers such as *SPRR2A*, *KRT16*, and *PI3*, while transcripts linked to mitochondrial and metabolic functions were among the most strongly repressed. Chromosomal mapping of Chromosomal mapping of DEGs revealed a genome-wide distribution without discernible clustering, implying that the expression shifts stem from broad regulatory disruptions rather than localized genomic alterations (**Figure 1D**).

**Figure 2:**
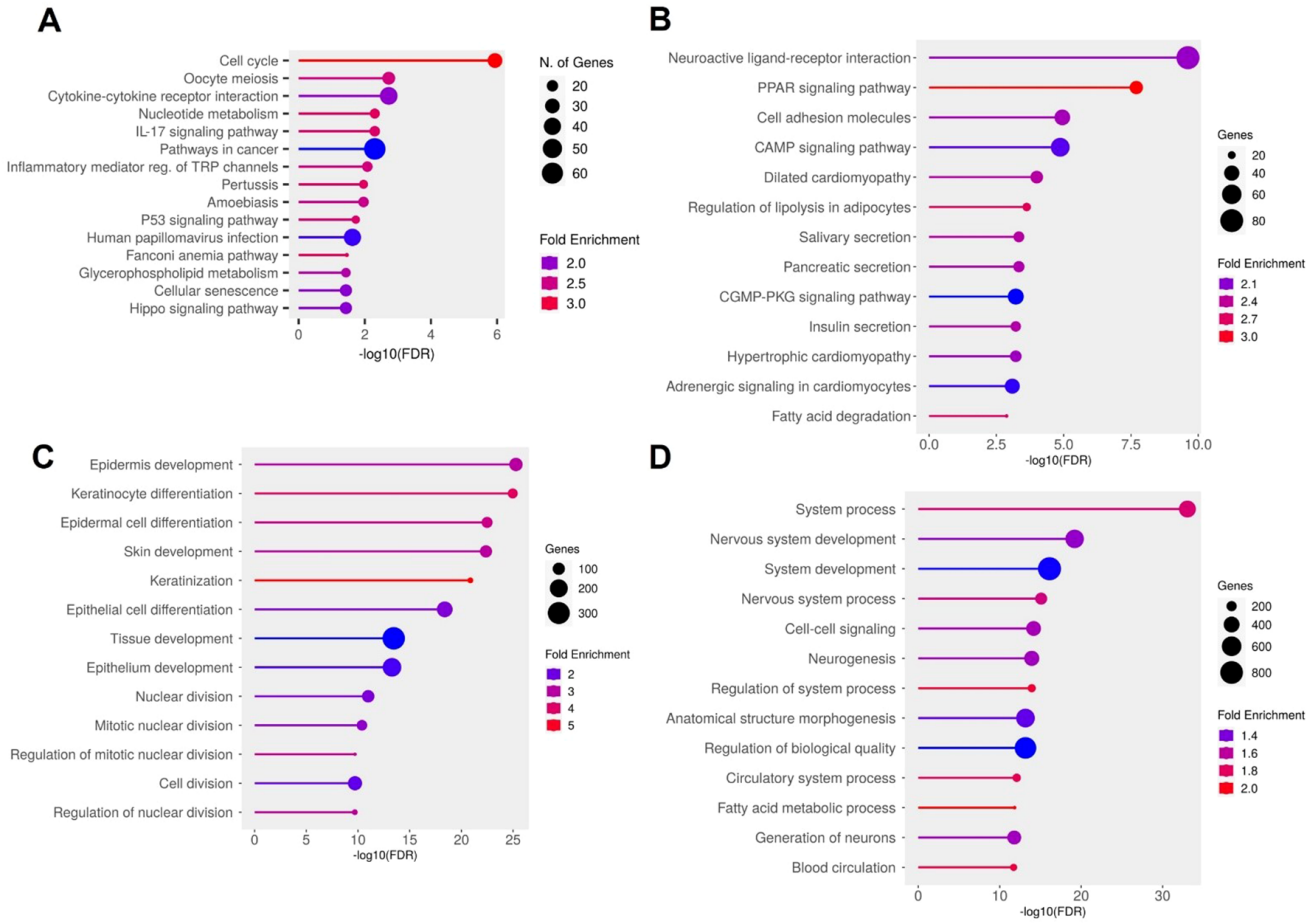
Functional enrichment analysis of differentially expressed genes (DEGs) in diabetic foot ulcer (DFU) skin samples. **(A, B)** KEGG pathway enrichment analysis. **(A)** Top enriched pathways in DFU skin associated with upregulated genes (DFU-vs.-CTRL), indicating heightened inflammatory, proliferative, and senescence activities. **(B)** Top enriched pathways for downregulated genes in DFU skin, suggesting disruptions in metabolic regulation and tissue integrity. **(C, D)** Gene Ontology (GO) biological process enrichment analysis. **(C)** GO terms enrichment for upregulated genes in DFU skin, reflecting compensatory tissue remodeling and hyperproliferation. **(D)** GO terms enrichment for downregulated genes in DFU skin, pointing to impaired neurovascular signaling and loss of tissue homeostasis. In all panels, circle size represents gene count, and color indicates fold enrichment. The x axis shows the –log10 of the FDR–adjusted p value, highlighting the statistical significance of each enrichment.

Pathway enrichment analysis further uncovered two overarching biological patterns. Upregulated genes in DFU skin were significantly enriched in pro-inflammatory and proliferative KEGG pathways, including cell cycle, IL17 signaling, cytokine–receptor interaction, p53 signaling, and cellular senescence—consistent with the hyperproliferative and inflamed phenotype observed in chronic, non-healing ulcers (**Figure 2A**). GO analysis reinforced this observation, revealing strong enrichment for biological processes such as epidermal development, keratinocyte differentiation, keratinization, and tissue remodeling (**Figure 2C**). Conversely, downregulated genes were predominantly involved in maintaining metabolic stability and structural integrity. KEGG pathways such as PPAR signaling, cell adhesion molecules, and cAMP signaling were notably depleted (**Figure 2B**). GO terms related to neurovascular development, circulatory system function, and fatty acid metabolism were also significantly under-represented (**Figure 2D**), indicating compromised neurovascular support and metabolic imbalance within the DFU microenvironment. Together, these findings outline a dual transcriptional program in DFU skin, characterized by widespread repression of genes associated with lipid metabolism, cell adhesion, and neurovascular signaling, alongside strong induction of pathways governing inflammation, proliferation, and epidermal stress responses. This transcriptomic polarity may underlie the pathological features of chronic DFU—persistent inflammation, hyperkeratinized wound edges, and impaired tissue repair—and identifies distinct molecular circuits that may serve as targets for future therapeutic intervention.

### 3.3 Transcriptomic profiling and functional enrichment analysis of diabetic foot ulcer in fat tissue

**Figure 3:**
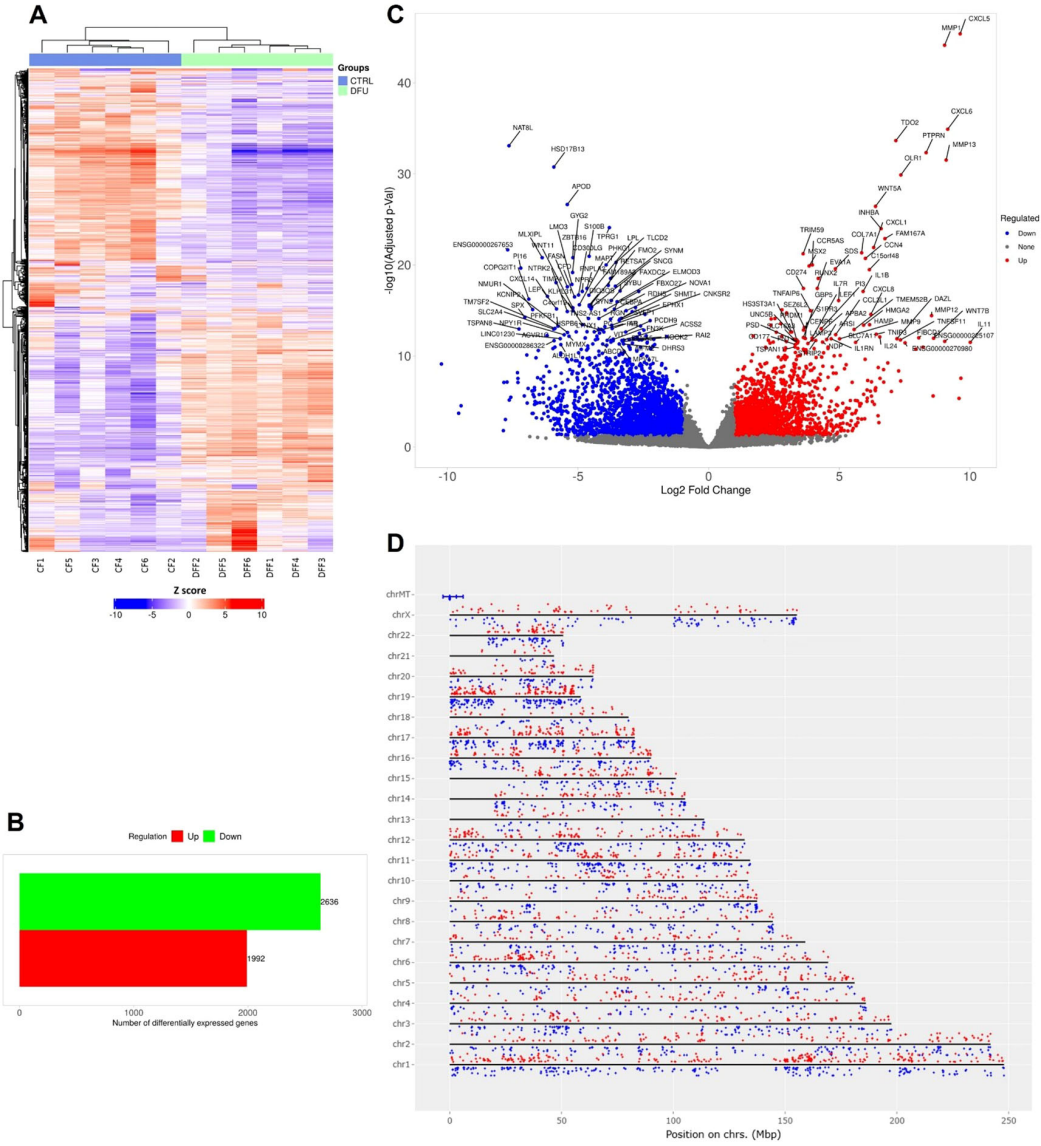
Gene expression analysis of diabetic foot ulcer in fat tissue. **(A)** Hierarchical clustering of transcript expression profiles showing distinct gene expression patterns between DFU and CTRL fat tissue samples. **(B)** Bar graph summarizing the number of differentially expressed genes (DEGs) in the DFU-vs.-CTRL fat tissue comparison. The red bar represents upregulated genes, and the green bar represents downregulated genes. **(C)** Volcano plot illustrating the distribution of DEGs. The x-axis shows log2 fold change, and the y-axis indicates –log10 of the adjusted p value. Red dots indicate significantly upregulated genes, blue dots indicate significantly downregulated genes, and gray dots represent non-significant changes. Highly significant genes are labeled. **(D)** Chromosomal mapping of DEGs showing their genomic distribution across human chromosomes. Red dots denote upregulated genes, and blue dots denote downregulated genes, with their positions plotted according to genomic coordinates and log2 fold change.

Transcriptomic profiling of DFU versus CTRL fat tissues revealed substantial differential gene expression patterns. Hierarchical clustering distinctly differentiated DFU and CTRL samples, reflecting marked transcriptional changes associated with DFU pathology (**Figure 3A**). Differential expression analysis (log2 fold change >1, adjusted p <0.05) identified 4,630 significantly dysregulated transcripts—1,992 of which were upregulated and 2,638 downregulated in DFU adipose tissue (**Figure 3B**; **Supplementary Table S2**). The volcano plot (**Figure 3C**) showed effect sizes spanning nearly ten-fold in both directions. Immune-related genes, including *MMP9*, *CXCL5*, and *IL1RN*, were among the most strongly upregulated, while key metabolic regulators involved in adipocyte function, such as *PPARGC1A*, *PLIN1*, and *ADIPOQ*, were prominently downregulated. Chromosomal distribution of these DEGs revealed a broad genomic dispersion across all autosomes and the X chromosome, with no evident clustering, suggesting a global reprogramming of transcription rather than localized genomic alterations (**Figure 3D**).

**Figure 4:**
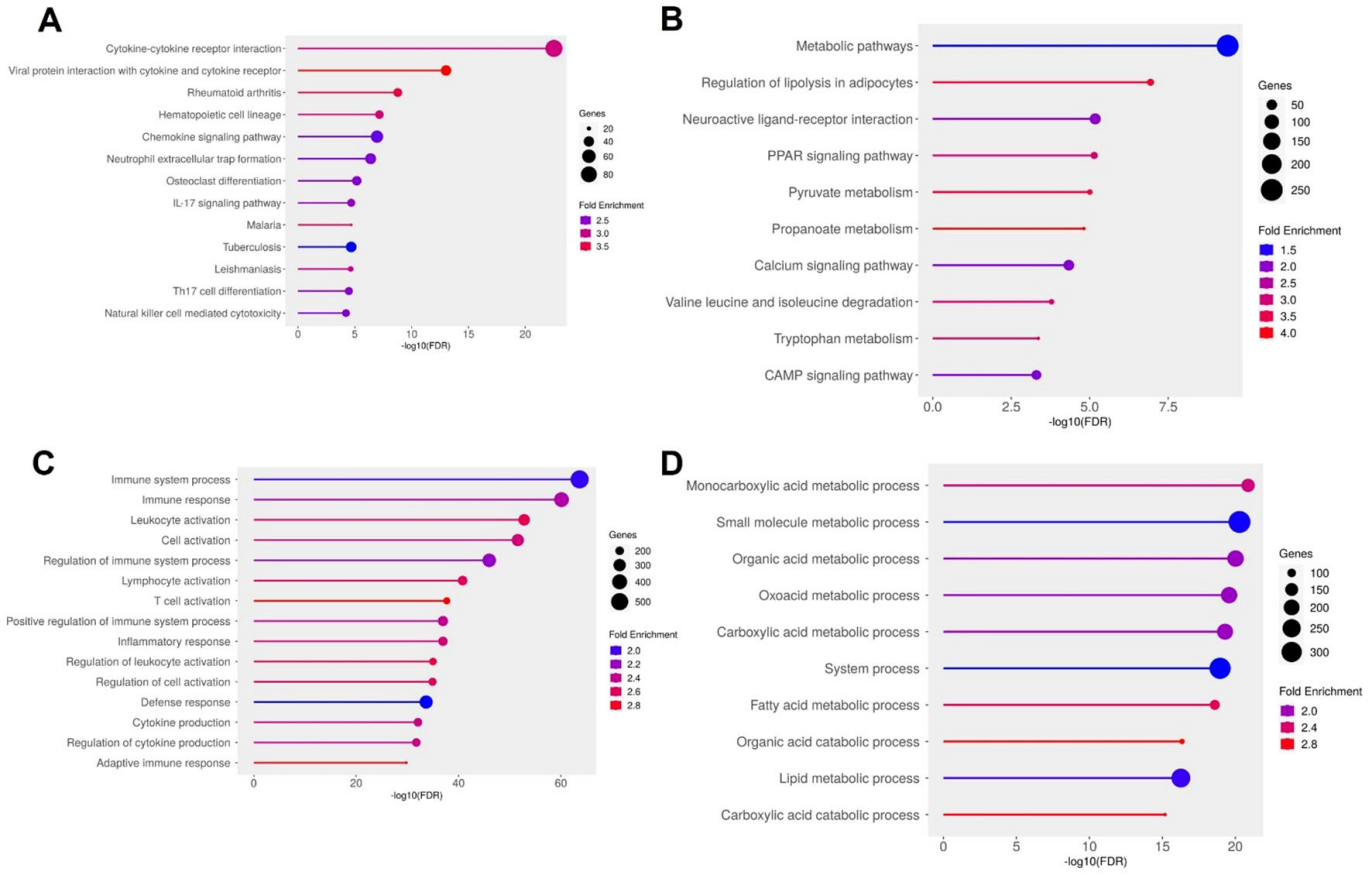
Functional enrichment analysis of differentially expressed genes (DEGs) in diabetic foot ulcer (DFU) fat samples. **(A, B)** KEGG pathway enrichment analysis. **(A)** Top enriched pathways in DFU fat associated with upregulated genes (DFU-vs.-CTRL), indicating enhanced immune activation, inflammatory signaling, and leukocyte-mediated responses. **(B)** KEGG pathways enriched among downregulated genes in DFU fat, highlighting disruptions in lipid metabolism, energy homeostasis, and neuroactive signaling. **(C, D)** Gene Ontology (GO) biological process enrichment analysis. **(C)** GO terms enriched for upregulated genes in DFU fat reveal activation of immune system processes, leukocyte recruitment, and pro-inflammatory responses. **(D)** GO enrichment for downregulated genes in DFU fat shows widespread suppression of metabolic processes, especially those involving fatty acid and carboxylic acid metabolism, suggesting metabolic dysfunction. In all panels, circle size represents the number of genes involved in each term, and color indicates the fold enrichment. The x-axis shows the –log10 of the FDR-adjusted p value, reflecting the statistical significance of each enriched term.

Pathway enrichment analysis supported this dichotomous expression landscape. KEGG analysis of the upregulated genes highlighted central inflammatory pathways, including cytokine–cytokine receptor interaction, *IL17* signaling, chemokine signaling, and neutrophil extracellular trap (NET) formation, indicating heightened immune activity within the ulcer environment (**Figure 4A**). Consistently, GO terms such as “immune system process”, “leukocyte activation”, “adaptive immune response”, and “cytokine production” were significantly overrepresented (**Figure 4C**), pointing to robust recruitment and activation of both innate and adaptive immune cells. In stark contrast, downregulated genes were enriched for pathways governing energy metabolism and lipid handling. KEGG terms included overarching metabolic processes as well as specific pathways like PPAR signaling, adipocyte lipolysis regulation, and degradation of branched-chain amino acids such as valine, leucine, and isoleucine (**Figure 4B**). GO analysis further supported this metabolic repression, with significant depletion of terms associated with fatty acid metabolism, monocarboxylic acid catabolism, and small molecule processing (**Figure 4D**). These findings describe an adipose environment where metabolic and lipid storage functions are transcriptionally silenced, while inflammatory and immune cell recruitment pathways are strongly activated. Together, these results illustrate a DFU adipose transcriptome defined by the co-existence of metabolic suppression and immunological overdrive. This dual state likely contributes to the chronic wound phenotype: impaired energy metabolism may hinder tissue repair, while sustained leukocyte infiltration and cytokine signaling exacerbate inflammation and tissue damage. These insights highlight both inflammatory pathways and adipocyte metabolic regulators as potential therapeutic targets for restoring homeostasis in DFU-affected tissues.

### 3.4 Transcriptomic profiling and functional enrichment analysis of diabetic foot ulcer in muscle tissue

**Figure 5:**
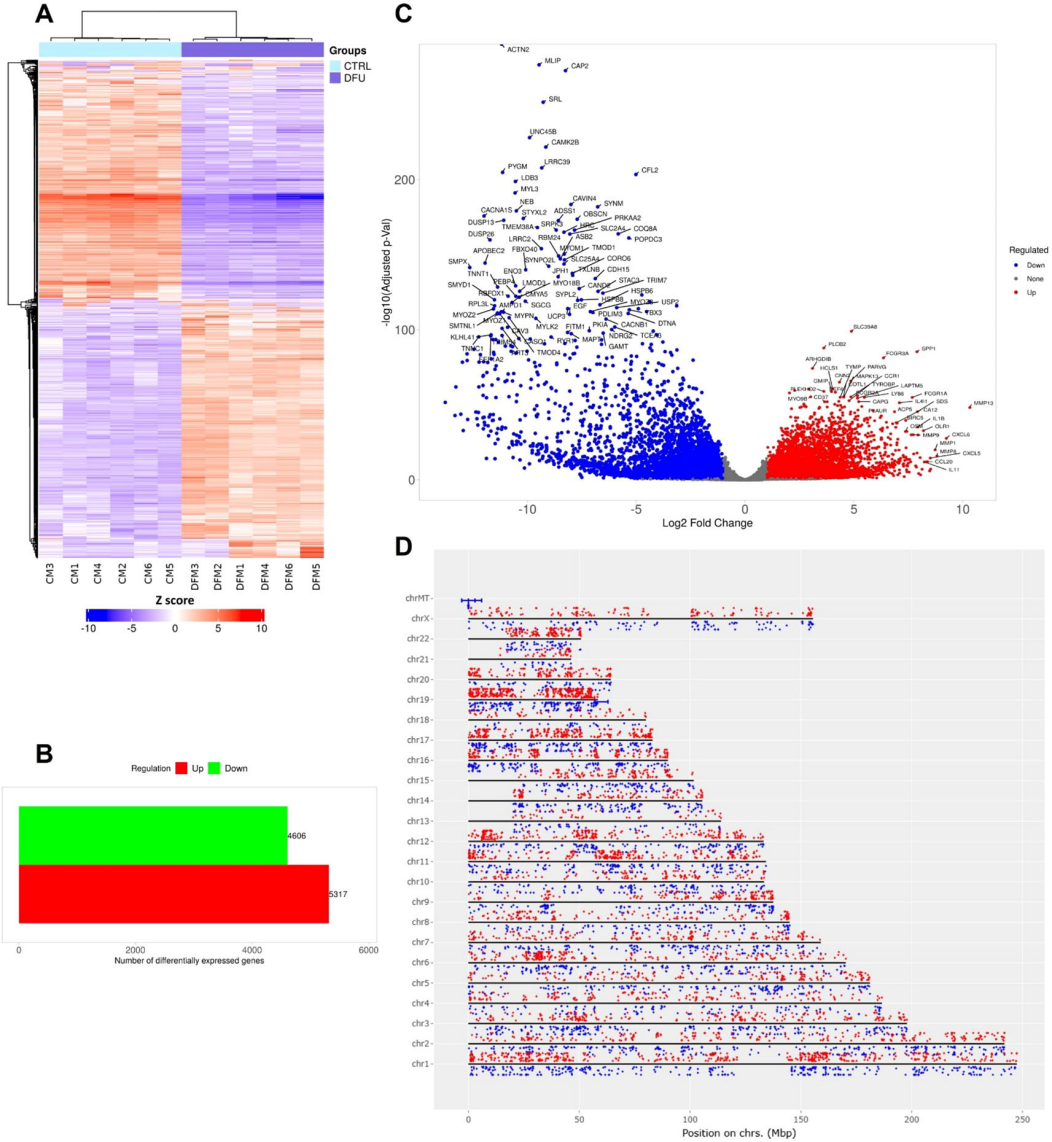
Differential expression profiling of diabetic foot ulcer in muscle tissue. **(A)** Hierarchical clustering heatmap of transcript expression for the top differentially expressed genes (DEGs) across the DFU and control (CTRL) muscle samples. Samples cluster cleanly by group, with most genes showing reciprocal up- versus down-regulation between CTRL and DFU. **(B)** Bar chart summarizing the total number of DEGs in DFU versus CTRL. **(C)** Volcano plot displaying DEGs between DFU and CTRL samples. The x-axis shows log2 fold-change (FC), and the y-axis shows -log10 (adjusted p value). Red dots represent significantly upregulated genes, blue dots represent significantly downregulated genes, and gray dots represent non-significant changes. Top genes with high fold changes and significance are labeled. **(D)** Genomic distribution of DEGs mapped across human chromosomes. Red dots represent upregulated genes, and blue dots represent downregulated genes. The distribution of dots around the chromosome corresponds to the log2 FC values.

To characterize molecular alterations in DFU muscle, we performed RNA-seq on CTRL and DFU patients. Hierarchical clustering of the top DEGs revealed clear separation of DFU and CTRL samples (**Figure 5A**), with reciprocal patterns of up- and down-regulation across groups. In total, 9,923 genes met significance thresholds (log2 FC >1, adjusted p <0.05), of which 5,317 were upregulated and 4,606 downregulated in DFU muscle relative to controls (**Figure 5B**; **Supplementary Table S3**). A volcano plot illustrated the magnitude and significance of these changes (**Figure 5C**). Notably, immune-related mediators such as *CXCL5*, *MMP9* and *CCL20* dominated the positive fold-change tail, whereas canonical myofibrillar and oxidative metabolism genes such as *ACTN2*, *MLIP*, *CAP2*, and numerous electron-transport components occupied the negative extreme (**Figure 5C**). Genome-wide mapping of DEGs further underscored a widespread transcriptional reprogramming in DFU muscle, with up- and down-regulated genes distributed across all autosomes and the mitochondrial chromosome (**Figure 5D**).

**Figure 6:**
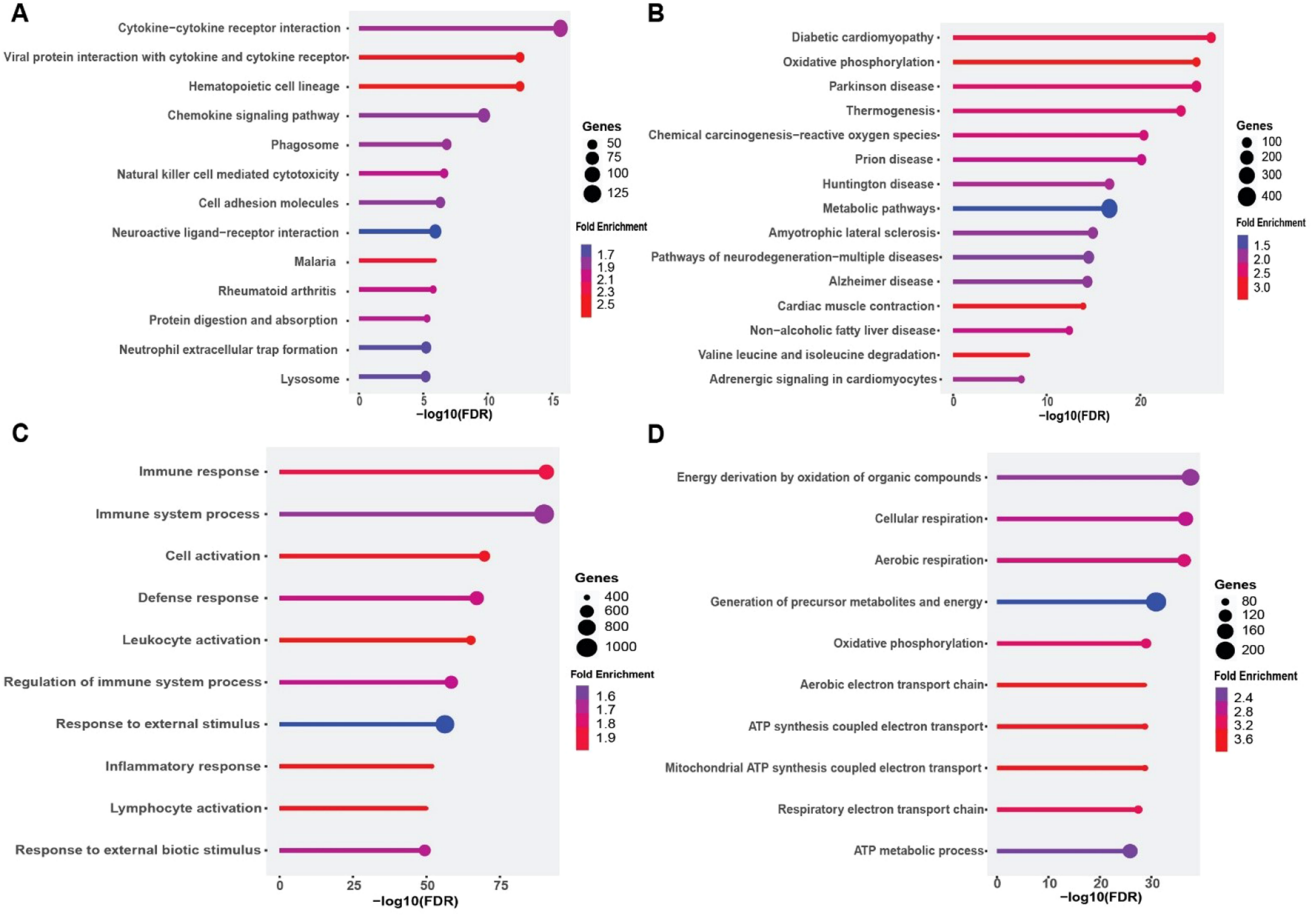
Functional enrichment analysis of differentially expressed genes (DEGs) in diabetic foot ulcer (DFU) muscle samples. **(A, B)** KEGG pathway enrichment analysis. **(A)** Top enriched pathways in DFU muscle associated with upregulated genes (DFU-vs.-CTRL), revealing activation of immune and inflammatory signaling pathways, including cytokine interactions and phagosome formation. **(B)** Enriched KEGG pathways for downregulated genes in DFU muscle, indicating significant impairments in mitochondrial function, energy metabolism, and neurodegenerative pathways. **(C, D)** Gene Ontology (GO) biological process enrichment analysis. **(C)** GO terms enriched among upregulated genes in DFU muscle highlight enhanced immune activation, leukocyte recruitment, and inflammatory responses. **(D)** GO enrichment for downregulated genes shows suppressed oxidative phosphorylation and aerobic respiration processes, pointing to mitochondrial dysfunction and reduced energy production capacity. In all panels, circle size reflects the number of genes involved, and color represents the fold enrichment. The x-axis shows the –log10 of the FDR-adjusted p value, indicating the statistical significance of each enriched term.

To determine the biological processes underlying these expression changes, we conducted KEGG and GO enrichment analyses separately for up- and down-regulated gene sets (**Figure 6**). Among upregulated genes, the highest enrichment was seen for cytokine–cytokine receptor interaction, viral protein interaction with cytokine receptors, and chemokine signaling pathways (**Figure 6A**). Complementary GO analysis highlighted robust activation of immune and inflammatory processes, including “immune response”, “leukocyte activation”, and “inflammatory response” (**Figure 6C**), reflecting a potent pro-inflammatory milieu in DFU muscle.

Conversely, downregulated genes were strongly enriched for pathways related to oxidative phosphorylation, Parkinson’s and Alzheimer’s diseases (which share mitochondrial dysfunction signatures), and thermogenesis (**Figure 6B**). GO terms such as “oxidative phosphorylation”, “ATP synthesis coupled electron transport”, and “respiratory electron transport chain” were among the most significantly depleted biological processes (**Figure 6D**). These findings indicate that the DFU muscle exhibits coordinated downregulation of genes governing mitochondrial bioenergetics, concomitant with upregulation of inflammatory networks. Collectively, these data reveal a dual transcriptional phenotype in DFU muscle characterized by heightened inflammation and immune activation alongside suppression of mitochondrial energy metabolism, which may contribute to impaired muscle function and wound healing in diabetic foot ulcers.

### 3.5 Shared and tissue-specific transcriptomic signatures of diabetic foot ulcer across skin, fat, and muscle tissues

**Figure 7:**
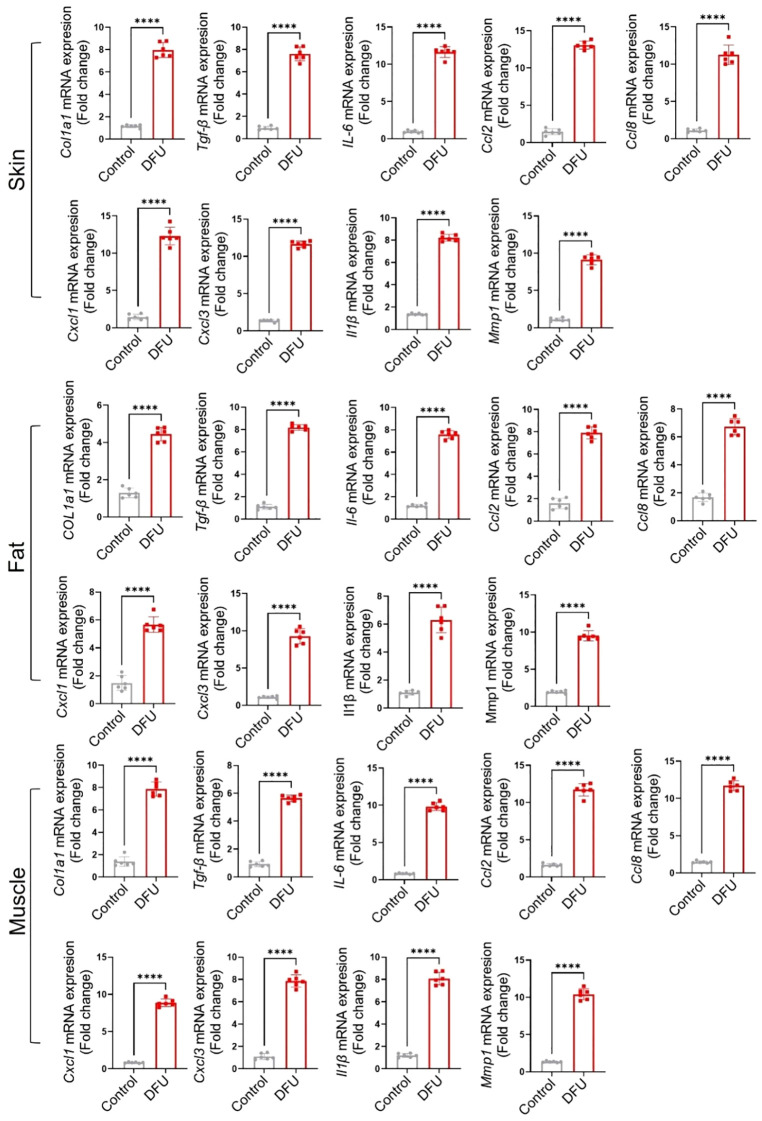
Validation of gene expression by qPCR of some highly expressed genes across the tissues. The name of each marker is written along the Y-axis.

**Figure 8:**
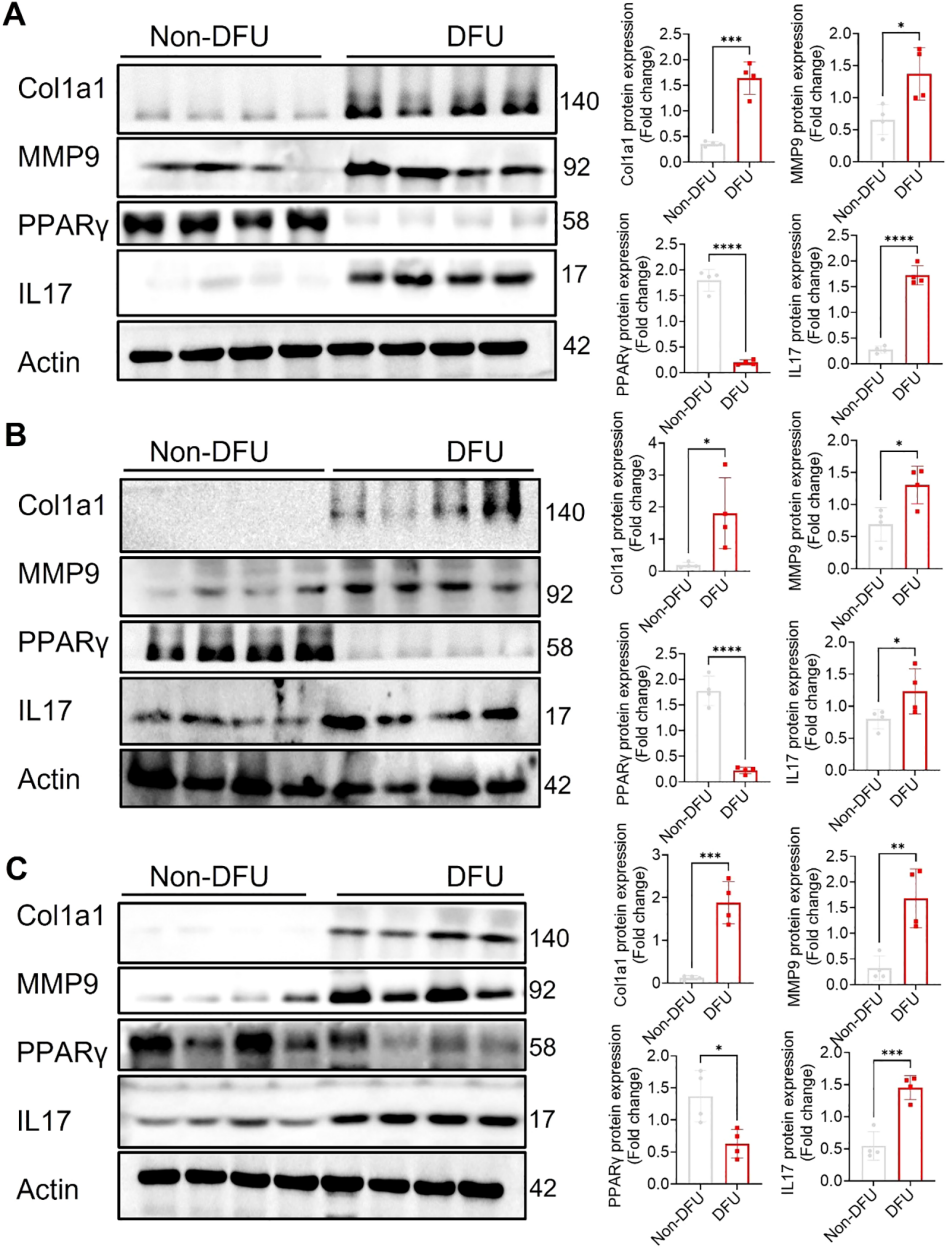
Validation of protein expression by Western blot of some selected proteins from top enriched pathways across the tissues. Representative western blots and quantitative analyses of Col1a1, MMP9, PPARγ, and IL17 expression in skin **(A)**, adipose **(B)**, and muscle **(C)** tissues from Non-DFU controls and DFU samples. Actin served as internal reference. Densitometric quantification is shown on the right as fold change relative to CTRL. DFU tissues show significantly increased expression of Col1a1, MMP9, and IL17, along with reduced expression of PPARγ, compared to Non-DFU controls. Data are presented as mean ± SEM; *p <0.05, **p <0.01, ***p <0.001, ****p <0.0001.

Transcriptomic profiling of DFU tissues revealed both shared and tissue-specific alterations across skin, fat, and muscle compartments (**Supplementary Tables S1–S3**; **Additional Files 1–3**), underscoring the complex and multifactorial nature of impaired wound healing in diabetes. Common themes across all three tissues included upregulation of immune and inflammatory pathways and downregulation of genes involved in metabolic homeostasis, mitochondrial function, and structural integrity. Some highly expressed genes were also validated through qPCR (**Figure 7**), further confirming the findings from the RNA-seq analysis. Western blot analysis (**Figure 8**) of skin, adipose, and muscle tissues revealed that COL1A1, MMP, and IL17 proteins were consistently upregulated in DFU samples compared with non-DFU controls, whereas PPARγ protein expression was reduced across all tissue types, with the most pronounced decrease observed in adipose tissue and skin. Together, these findings validate the protein-level confirmation of RNA-seq results.

#### 3.5.1 Shared upregulated transcriptomic signatures

A prominent feature across skin, fat, and muscle tissues was the consistent upregulation of genes involved in inflammatory and immune responses. Chemokines (e.g., *CXCL1*, *CXCL3*, *CXCL5*, *CXCL8*, *CXCL13*, *CCL2*, *CCL7*, *CCL8*, *CCL20*) and their receptors (e.g., *CCR2–5*, *CXCR1–4*) were universally elevated, supporting a model of sustained leukocyte recruitment and chronic immune activation in DFUs. Pro-inflammatory cytokines, including *IL1B*, *IL6*, *IL10*, *IL19*, *IL24*, and members of the TNF and TLR families (e.g., *TNFRSF1B*, *TNFAIP3*, *TLR2*, *TLR4*) were also upregulated in all three tissues, indicating broad activation of both innate and adaptive immune pathways. The induction of NF-κB signaling components (e.g., *NFKB1*, *NFKB2*, *RELB*) and related adaptors (e.g., *MYD88*, *TRAF1*) further supports systemic inflammatory activation. Additionally, all tissues showed upregulation of genes related to extracellular matrix (ECM) remodeling and fibrotic signaling, including integrins (e.g., *ITGAL*, *ITGA4/5*, *ITGB2/3*), adhesion molecules (*ICAM1*, *VCAM1*, *PECAM1*), matrix proteins (*FN1*, *COL1A1*, *COL6A1*), and fibrotic regulators (*TGFB1*, *THBS1*). These findings suggest a persistent reparative or fibrotic state in DFUs, possibly contributing to scar formation and impaired tissue function.

In the skin (**Supplementary Table S1**; **Additional File 1**), upregulation was skewed toward cell cycle and mitotic regulators (e.g., *CDK1*, *CCNA2*, *CCNB1/2*, *PLK1*, *FOXM1*), reflecting hyperproliferative responses likely driven by chronic injury. These were accompanied by elevated expression of apoptosis- and senescence-associated genes (e.g., *TP53I3*, *CHEK1*, *CDKN2B*), suggesting an environment of both regeneration and stress-induced arrest. The presence of senescence-associated secretory phenotype (SASP) markers, such as *IL1A*, *IL1B*, and *CXCL8*, indicated a pro-inflammatory milieu perpetuated by senescent cells.

#### 3.5.2 Tissue-specific upregulated transcriptomic signatures

In fat tissue (**Supplementary Table S2**; **Additional File 2**), upregulated genes highlighted heightened immune cell recruitment and cytokine signaling. Adipose tissue demonstrated a strong increase in chemokine ligands and receptors, interleukin signaling (*IL1A/B, IL6R, IL18*), TNF superfamily members (e.g., *TNFRSF9, TNFRSF11*), and apoptosis-associated markers (e.g., *FAS, FASLG*). Importantly, extracellular matrix proteases such as *MMP1/3/9/13* were upregulated, reflecting a proteolytic environment that may impede matrix stability and tissue repair. Fat tissue also exhibited activation of hypoxia (HIF1A) and immune transcription factors (*IRF1, JAK3, FOXP3*), indicating systemic stress and immune dysregulation.

In the muscle (**Supplementary Table S3**; **Additional File 3**), transcriptomic changes were dominated by immune activation, ECM remodeling, and apoptotic signaling. Numerous adaptive immune markers (*CD3D, CD4, CD19*), co-stimulatory molecules (*CD40, CD80, ICOS*), and macrophage/neutrophil markers (*MPO, LAMP1, CTSD, CTSS*) were upregulated. This was accompanied by increased fibrotic gene expression (*COL1A1, SPP1, LAMA1*) and pro-apoptotic genes (*CASP3, FAS*). Upregulation of lysosomal genes (*ATP6V0D2, ATP6V1B2*) further reflected enhanced phagocytic and degradative activity.

#### 3.5.3 Shared downregulated transcriptomic signatures

A shared signature of downregulation across all tissues involved mitochondrial and metabolic dysfunction. Key regulators of fatty acid oxidation (e.g., *ACADM, ACADL, HADHA, ECHS1*), TCA cycle components (e.g., *IDH2, MDH2, SDHA*), and oxidative phosphorylation subunits (e.g., *NDUFS7, COX4I1, ATP5F1A*) were suppressed in both muscle and fat, and to a lesser extent in skin. These findings indicate energy deprivation and metabolic inflexibility in DFUs, consistent with the observed clinical hypoxia and poor healing capacity. Also common was the downregulation of insulin signaling and glucose metabolism genes such as *IRS1, PIK3R1, AKT2*, and *PRKAA2* across muscle, fat, and skin, suggesting local insulin resistance and impaired metabolic responsiveness. Proteostasis and oxidative stress response genes (e.g., *SOD1, CAT*, *BECN1*) were also consistently reduced, pointing to diminished ability to clear damaged proteins and organelles.

#### 3.5.4 Tissue-specific downregulated transcriptomic signatures

In the skin, suppressed genes included those involved in lipid metabolism (e.g., *PPARG, FABP4, PLIN1–5*) and hormonal signaling (e.g., *PRLR, LEP, VEGFB/D*), indicating impaired energy use and paracrine signaling. Notably, many genes related to epidermal structure and barrier function (e.g., CLDN family, *ITGA7/8/9, SGCG*) were downregulated, suggesting compromised skin integrity.

In the fat, broad downregulation of lipolytic enzymes genes (e.g., *PNPLA2, MGLL*), fatty acid transporters (*SLC27A1/2*), and thermogenic regulators (e.g., *RXRA, RXRG*) revealed metabolic silencing and dedifferentiation of adipocytes. A decline in neuroendocrine and angiogenic signals (e.g., *VEGFB, FGF10, LEPR*) suggested impaired systemic cross-talk necessary for wound healing.

In the muscle, downregulation extended to genes critical for contractile function (*MYH7, TNNT2, TPM1–3*), calcium handling (*ATP2A1, RYR1*), and sarcomeric integrity (*DMD, SGCA-D*). Downregulation of mitochondrial biogenesis regulators (e.g., *PPARGC1A, PRKAA2*) and proteasomal components further highlighted disrupted muscle homeostasis and regeneration. Additionally, repression of autophagy markers (e.g., *MAP1LC3B, ULK1*) suggested reduced cellular cleanup mechanisms essential for myofiber maintenance.

Together, the transcriptomic signatures across skin, fat, and muscle tissues of DFU patients reveal a unified pathological landscape marked by chronic inflammation, disrupted energy metabolism, immune dysregulation, and tissue remodeling. While some gene expression changes reflect compensatory responses to injury, such as cell proliferation, angiogenesis, or cytokine signaling—the widespread downregulation of metabolic, structural, and regenerative pathways highlights the systemic failure of these tissues to resolve damage and initiate effective repair. Tissue-specific patterns further underscore the unique vulnerabilities of each compartment, with skin showing mitotic dysregulation and senescence, fat exhibiting metabolic dedifferentiation and immune infiltration, and muscle displaying fibrotic remodeling and mitochondrial collapse. These findings provide critical insight into the molecular underpinnings of chronicity and poor healing in diabetic foot ulcers.

### 3.6 Tissue cellular composition and inter-tissue crosstalk in DFUs

**Figure 9:**
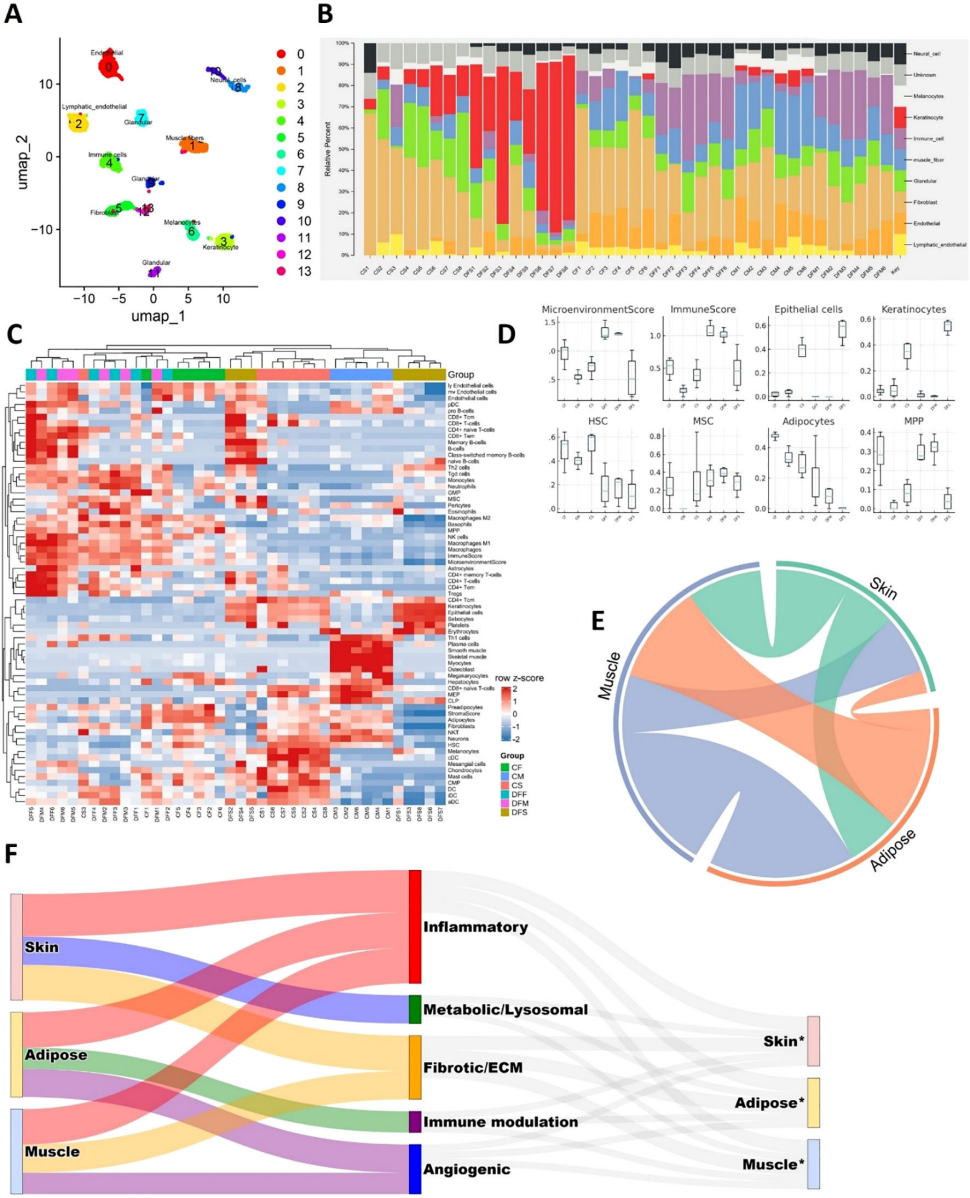
Tissue cellular composition and cross-talk in DFUs. **(A)** Single-cell UMAP visualization of the custom reference matrix derived from publicly available single-cell atlases, used for computational deconvolution of bulk RNA-seq profiles. **(B)** Major cell type composition across bulk RNA mixtures, estimated using CIBERSORTx and xCell. **(C)** Heatmap showing detailed deconvolution signatures, highlighting relative contributions of diverse stromal, immune, and parenchymal populations in skin, adipose, and muscle. **(D)** Boxplots showing refined enrichment scores of representative cell classes, including microenvironmental stromal cells, immune lineages, epithelial/keratinocytes, hematopoietic stem cells (HSCs), mesenchymal stem cells (MSCs), adipocytes, and multipotent progenitors (MPPs). **(E)** Chord diagram illustrating inter-tissue interactions among skin, adipose, and muscle, demonstrating extensive bidirectional crosstalk. **(F)** Sankey diagram summarizing paracrine ligand–receptor networks across tissue compartments. Left: sender tissues; middle: functional categories of ligands; right: receiver tissues (*).

Computational deconvolution of bulk RNA-seq profiles revealed distinct alterations in cellular composition across DFU and control tissues (**Figure 9**). Major cell type distributions across samples (**Figure 9B**) indicated that skin (CTRL skin; CS vs. diabetic foot skin;DFS), adipose (CTRL fat; CF vs. diabetic foot fat; DFF), and muscle (CTRL muscle; CM vs. diabetic foot muscle; DFM) each exhibited distinct compositional shifts. Detailed resolution of immune, stromal, and parenchymal subtypes (**Figure 9C**) highlighted that DFS was enriched in keratinocytes, endothelial cells, and diverse immune subsets compared to CS, reflecting proliferative and inflammatory remodeling of the epidermal barrier. DFF showed expansion of stromal fibroblasts, macrophages, and mesenchymal stromal cells alongside diminished adipocyte fractions relative to CF, consistent with adipocyte dedifferentiation and pro-fibrotic remodeling. DFM samples exhibited loss of mature myofiber signatures and increased representation of progenitor and immune lineages compared to CM, suggesting myofiber attrition with stromal and immune infiltration. Boxplot analyses of representative cell classes (**Figure 9D**) corroborated these trends: higher epithelial and immune scores in DFS, elevated fibroblast and stromal signatures in DFF, and reduced contractile cell fractions with increased progenitor compartments in DFM. Collectively, these findings demonstrate that DFU tissues undergo compartment-specific shifts characterized by keratinocyte hyperplasia in skin, adipocyte depletion in adipose, and myofiber loss in muscle, each accompanied by expansion of stromal and immune components.

To explore how these compositional imbalances translate into inter-tissue communication, we inferred ligand–receptor interactions across skin, adipose, and muscle. A chord diagram highlighted extensive bidirectional signaling among all three compartments (**Figure 9E**), while a Sankey plot summarized the functional distribution of paracrine interactions across inflammatory, fibrotic/ECM, angiogenic, metabolic/lysosomal, and immune-modulatory categories (**Figure 9F**). Detailed analyses of direction-specific ligand–receptor axes are presented in **Supplementary Figures S4–S9**. To further probe inter-compartmental communication, we constructed tissue-resolved ligand–receptor expression maps by integrating differential expression with curated ligand–receptor databases and NicheNet prioritization. This analysis revealed a dense paracrine signaling network linking skin, adipose, and muscle, in which inflammatory, fibrotic, angiogenic, and metabolic cues were exchanged bidirectionally. Skin-derived ligands prominently included chemokines (*CXCL5*), cytokines (*OSM, TNFSF13B, IL36G*), and structural components (*COL6A1/3*, laminins, *POSTN*) (**Supplementary Figures S4, S6**). These signals projected into adipose and muscle receptors linked to immune activation, matrix remodeling, and stress pathways, indicating that inflamed skin actively drives inflammation, fibrosis, and metabolic dysfunction in deeper tissues. In turn, adipose and muscle secreted angiogenic and fibrotic ligands (ANGPT1/4, TGFB3, VEGFC, THBS2), immune modulators (CD276, PDCD1LG2, SIGLEC1), and cytotoxic mediators (GZMA, TNFSF11) (**Supplementary Figures S5, S7–S9**), which mapped to receptors in skin and adipose. Such reciprocal signaling suggests that stressed adipose and muscle compartments feed back into skin to reinforce keratinocyte dysfunction, aberrant angiogenesis, and chronic immune activation.

Across all tissue pairs, lysosomal and metabolic mediators (GRN, PSAP, DHCR24, RARRES2) emerged as recurrent ligands (**Supplementary Figures S4–S9**), highlighting that disrupted metabolism and degradative stress are communicated between compartments alongside classical inflammatory signals. Collectively, these findings reveal that DFUs are sustained by a bidirectional, multi-tissue paracrine network in which skin, adipose, and muscle amplify one another’s inflammatory, fibrotic, and metabolic dysfunction. This inter-tissue dialogue likely underlies the persistence of the non-healing wound environment.

## 4 Discussion

In this study, we conducted a comprehensive, multi-tissue transcriptomic analysis of DFUs across human skin, subcutaneous fat, and muscle. Our findings reveal both shared and tissue-specific molecular signatures that underpin chronic ulcer pathology. A consistent feature across all three tissue compartments was the robust and coordinated upregulation of immune and inflammatory pathways—characterized by elevated expression of chemokines, cytokines, and components of NF-κB signaling, along with mediators of ECM remodeling and fibrotic drivers. Simultaneously, genes involved in mitochondrial bioenergetics, fatty acid oxidation, and insulin signaling were uniformly downregulated, suggesting a profound metabolic collapse that may contribute to impaired wound healing and localized insulin resistance. This dual molecular phenotype, marked by “inflammatory overdrive” and “metabolic shutdown”, emerged as a defining transcriptomic hallmark of DFUs in this study. These results contribute to the growing transcriptomic atlas of DFUs, which is increasingly moving toward spatially resolved mapping (39, 40).

A particularly striking observation was the uniform amplification of chemokine–cytokine signaling networks, including *CXCL1/3/5/8, CCL2/7/8/20, IL1β*, and *IL6* alongside their upstream NF-κB adaptors across all tissues. Similar transcriptional signatures have been observed in single-cell studies of chronic wounds, where sustained chemokine activity perpetuates a feed-forward loop of neutrophil and macrophage recruitment (41, 42). Notably, excessive *IL1β* signaling has been functionally linked to impaired wound closure and localized insulin resistance in both human and murine DFUs (43, 44), reinforcing the pathological relevance of the inflammatory state we observed.

Among the inflammatory pathways, *IL17* signaling emerged as a top-ranking, shared mechanism. While transient *IL17A* activity can enhance early immune defense, sustained activation is known to prolong keratinocyte stress and ECM degradation, ultimately hindering re-epithelialization (45, 46). Recent studies have shown that modulation of the IL17A–mTOR–HIF-1α axis—via interventions such as fecal microbiota transplantation can accelerate diabetic wound closure in mice, highlighting this pathway as a potential therapeutic switch between acute and chronic inflammatory states (47). Our data further support the clinical rationale for selectively tempering rather than completely inhibiting *IL17* activity.

In parallel with immune amplification, we observed widespread suppression of PPAR-regulated lipid metabolism genes (e.g., *PPARG, ADIPOQ, PLIN1*) across all tissues. This pattern aligns with evidence that PPARγ downregulation impairs macrophage polarization and angiogenesis in diabetic wounds (48). Moreover, the concurrent repression of insulin signaling genes is consistent with clinical observations of local insulin resistance in DFUs. These findings suggest that restoring insulin sensitivity, potentially via PPAR agonists, could simultaneously resolve metabolic dysfunction and dampen inflammation (49, 50). Preclinical studies have shown that both topical and systemic PPARγ agonists can re-engage oxidative metabolism, suppress inflammation, and enhance wound closure (51, 52). The uniform PPAR deficit observed across tissues in our study suggests that activating this pathway could benefit not only the epidermis but also the underlying adipose and muscular compartments.

The most pronounced mitochondrial dysfunction was detected in muscle tissue, where transcripts related to oxidative phosphorylation (*NDUFS7, COX4I1, ATP5F1A*) were sharply downregulated. These findings support recent evidence that diabetes-induced mitochondrial-derived vesicles disrupt redox balance and impair wound repair (53), as well as previous observations that diabetic skeletal muscle exhibits defective regeneration due to sustained inflammation and impaired energy metabolism (54). These results highlight the potential therapeutic value of strategies aimed at restoring mitochondrial dynamics or enhancing biogenesis.

In the skin, we noted an intriguing co-expression of mitotic regulators (e.g., *CDK1, FOXM1*) with a senescence-associated secretory phenotype (SASP), characterized by elevated *IL1α/β* and *CXCL8*. The accumulation of senescent keratinocytes has been implicated in persistent inflammation and delayed wound healing in both aging and diabetic skin (55). Recent studies support the use of senolytic and SASP-modulating agents as adjuvant treatments for DFUs (6, 11), a rationale that is strengthened by our findings.

Although the inflammatory–metabolic axis was a shared feature, each tissue compartment exhibited distinct mal-adaptations. The skin displayed downregulation of barrier and lipid metabolism genes, potentially explaining the hyper-keratinized yet dysfunctional wound edge characteristic of chronic ulcers. Adipose tissue showed signs of adipocyte dedifferentiation and upregulation of matrix proteases, potentially destabilizing the extracellular scaffold required for granulation. Muscle tissue exhibited evidence of fibrotic remodeling superimposed on mitochondrial suppression, factors that likely impair pressure tolerance and contribute to ulcer recurrence. These compartment-specific alterations emphasize that while inflammation and metabolic dysfunction are common threads, the underlying mechanisms of chronicity differ by tissue and may require tailored therapeutic approaches.

Our integrative analysis of tissue composition and inter-tissue signaling in DFUs highlights that chronic ulceration is not confined to local changes within a single tissue, but rather emerges from coordinated disruptions across skin, adipose, and muscle compartments. The deconvolution-based cellular profiling revealed compartment-specific alterations—keratinocyte hyperplasia and immune infiltration in skin (56), stromal expansion and adipocyte depletion in adipose, and myofiber attrition with progenitor enrichment in muscle (57)—that collectively point to a microenvironment characterized by proliferative stress, fibrotic remodeling, and impaired regenerative capacity. Importantly, these compositional shifts were coupled with a dense network of paracrine ligand–receptor interactions spanning all three tissues. Inflammatory chemokines and cytokines secreted by skin projected strongly into adipose and muscle, while adipose and muscle reciprocated with fibrotic, angiogenic, and immunomodulatory ligands feeding back into skin (58–60). The recurrent presence of metabolic and lysosomal mediators across tissue pairs further suggests that metabolic stress is propagated alongside immune and structural remodeling signals, reinforcing the chronicity of DFUs. These findings advance the view that DFUs represent a systems-level pathology in which inter-compartmental crosstalk perpetuates inflammation, fibrosis, and metabolic dysfunction. Targeting these cross-tissue signaling loops—rather than focusing solely on single-tissue pathways—may therefore be necessary to break the cycle of non-healing and restore wound resolution in diabetic patients.

However, our study’s cross-sectional design limits causal inference, providing only a static view of DFU pathology. The RNA-seq approach, while powerful, does not capture protein levels, post-translational modifications, or cellular spatial organization. Thus, future integration with proteomic, metabolomic, and spatial transcriptomic data will be crucial for a more comprehensive understanding. Longitudinal studies and functional perturbation experiments will also be necessary to validate key molecular drivers and therapeutic leverage points.

## 5 Conclusion

Our multi-tissue transcriptomic atlas defines DFUs as syndromic lesions arising from the convergence of chronic immune dysregulation and systemic metabolic collapse. By identifying both shared and tissue-specific molecular drivers, this study delivers critical insights into the underlying biology of chronic wounds. These findings offer a strategic foundation for the development of targeted, combinatorial therapies that address the dual burden of inflammation and metabolic dysfunction, thus advancing the goal of restoring effective tissue repair and improving outcomes for diabetic patients.

## Data Availability

The RNA-seq datasets generated during the current study have been deposited and are publicly accessible in the National Genomics Data Center (NGDC) under the project accession number PRJCA027046. The corresponding samples have been deposited under accession numbers SAMC3877250 to SAMC3877301, specifically including the following entries: SAMC3877250, SAMC3877252, SAMC3877254–SAMC3877260, SAMC3877263–SAMC3877270, SAMC3877272–SAMC3877275, SAMC3877277, SAMC3877279–SAMC3877280, SAMC3877282–SAMC3877285, SAMC3877287–SAMC3877290, SAMC3877292, SAMC3877294–SAMC3877295, and SAMC3877297–SAMC3877301.
