# Supplementary material for: Comprehensive transcriptomic profiling reveals tissue-specific molecular signatures and dysregulated pathways in human diabetic foot ulcers
Source: Front Endocrinol (Lausanne). 2025 Nov 3;16:1669205. doi: 10.3389/fendo.2025.1669205 (PMC12620202; doi:10.3389/fendo.2025.1669205)

**Additional file 3: The enriched KEGG pathways and gene mapping for muscle**

**Color key:** Red, up regulated genes; Green, down regulated genes


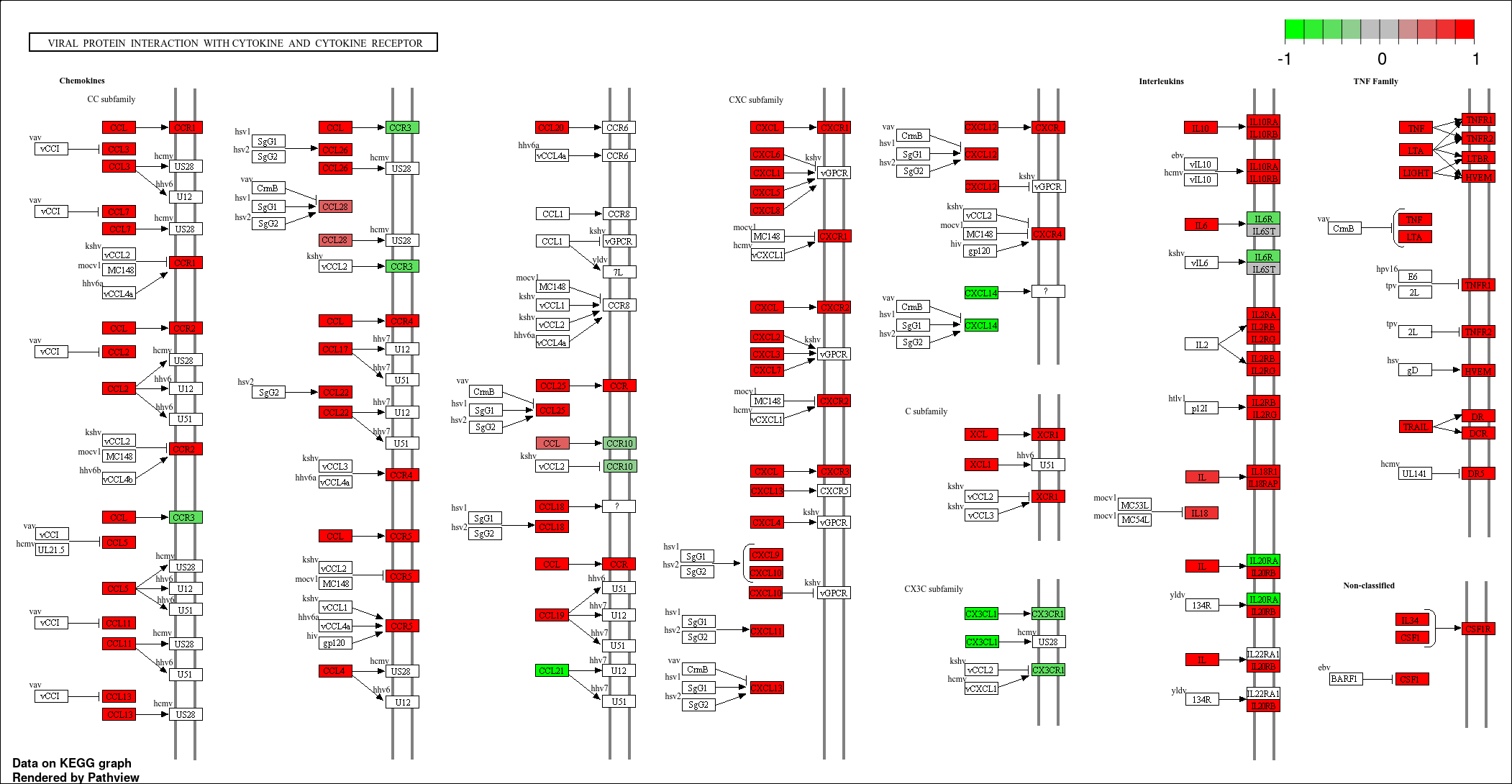


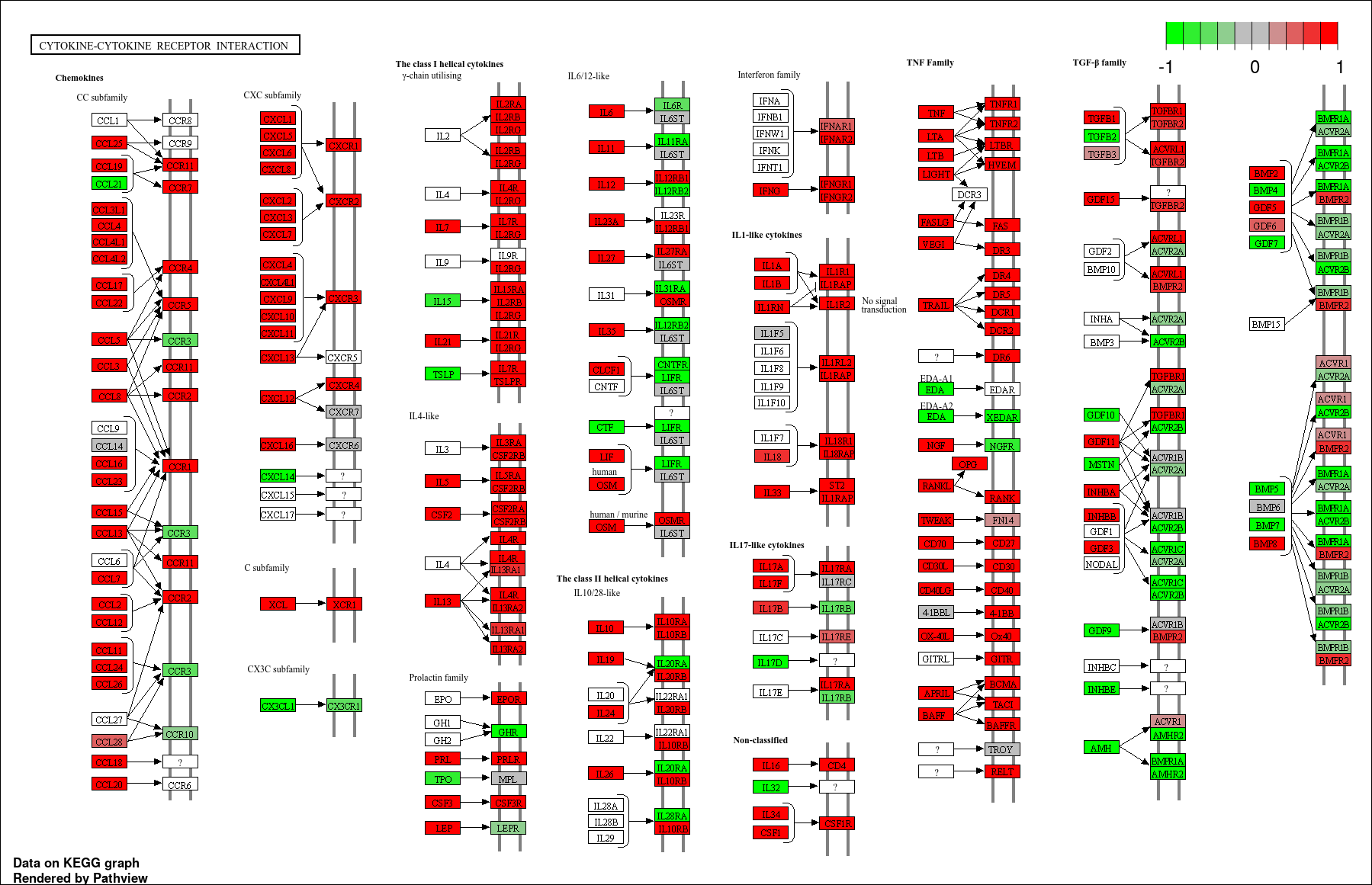


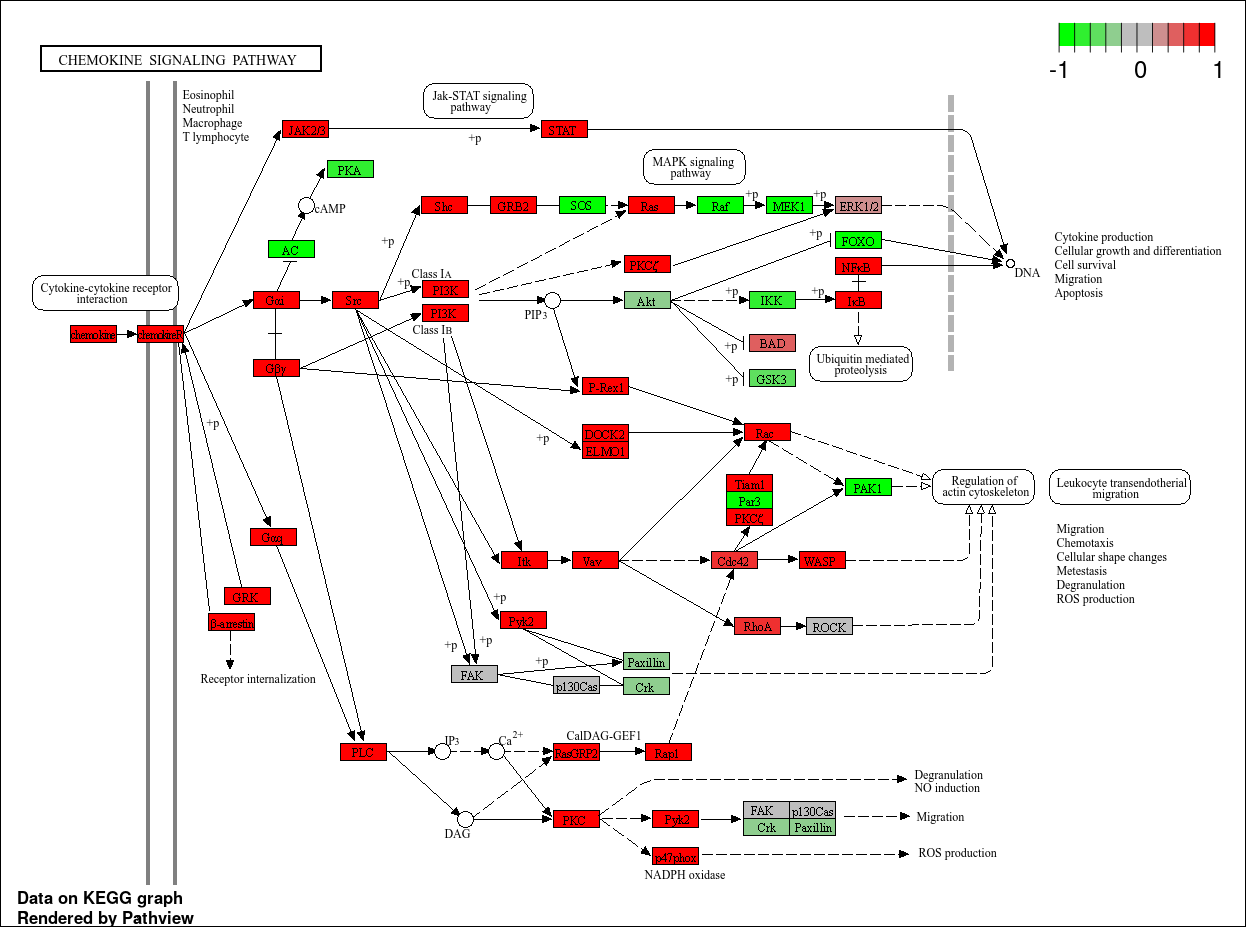


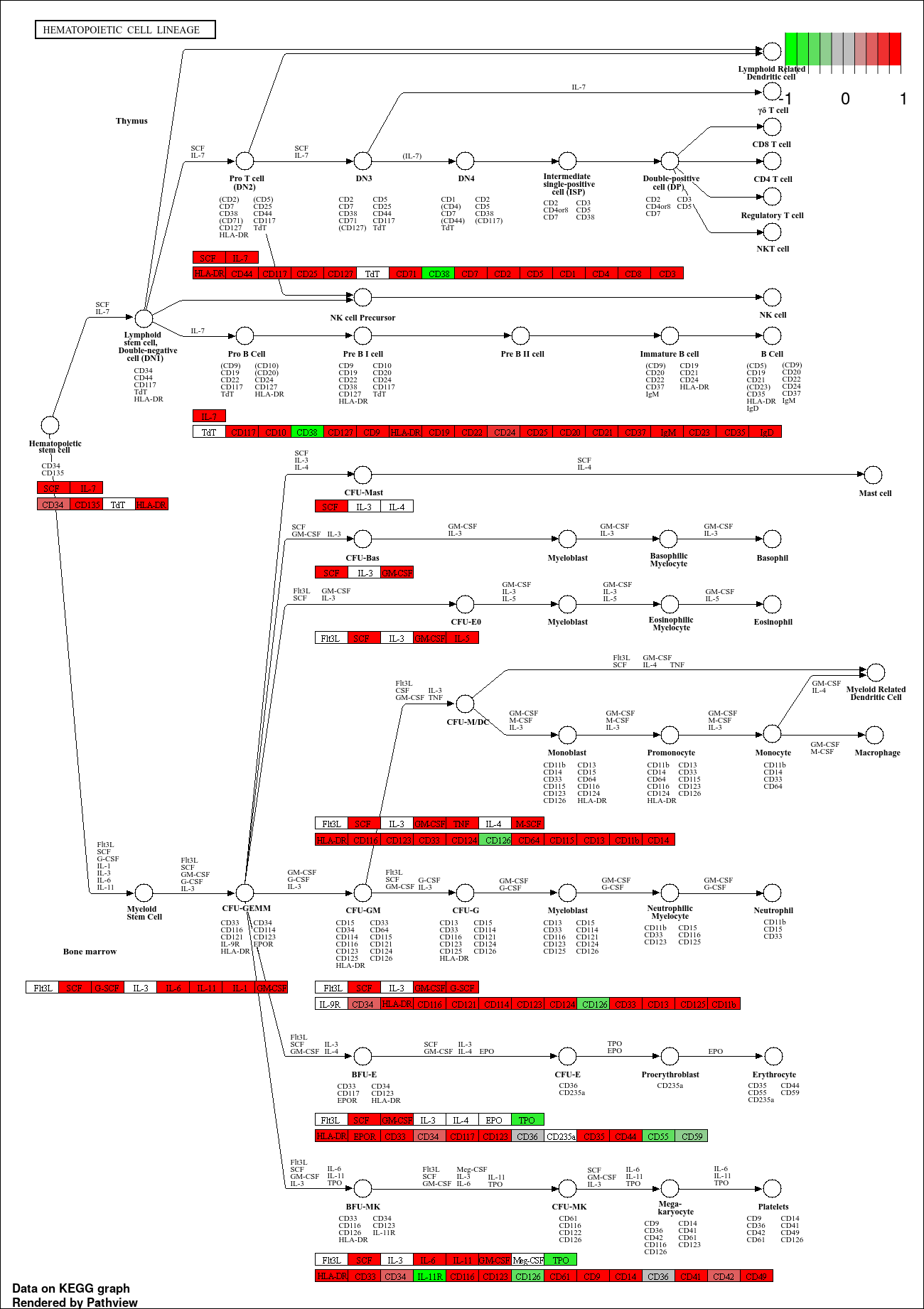


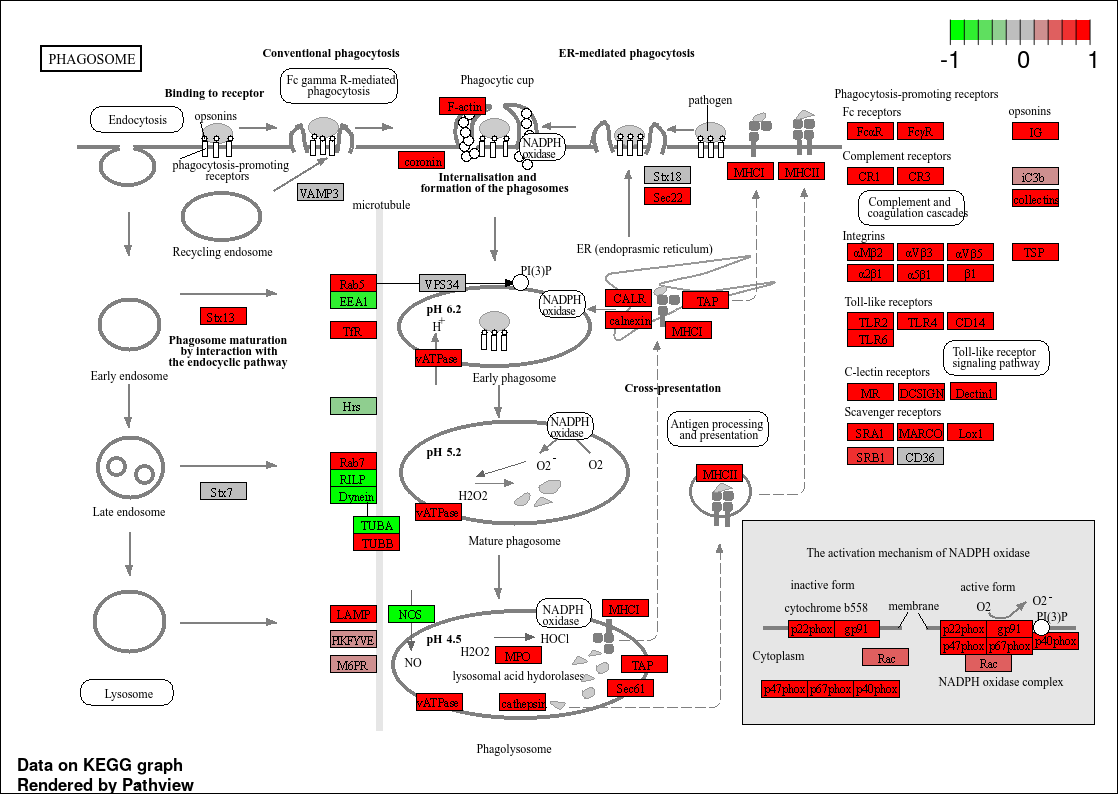


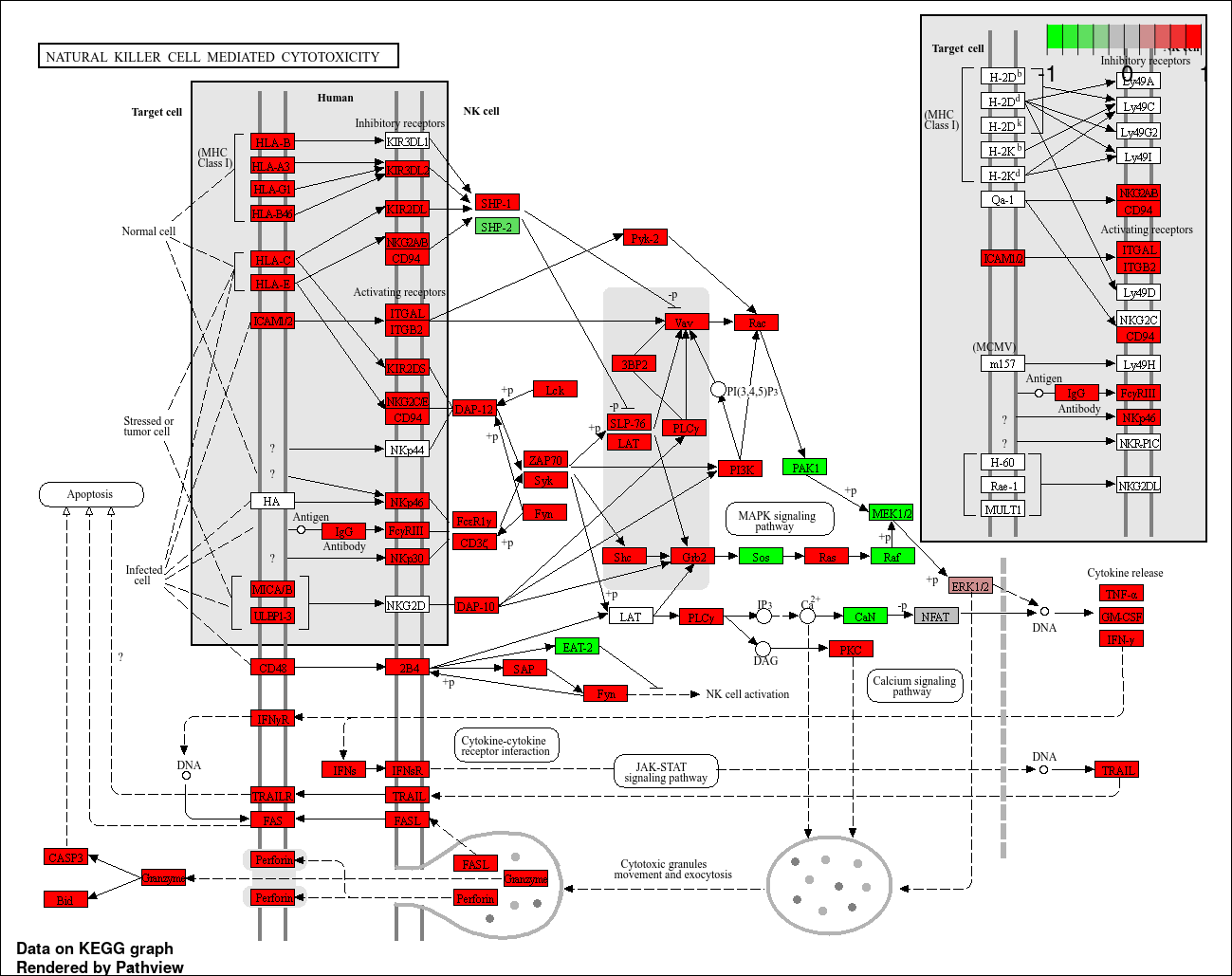


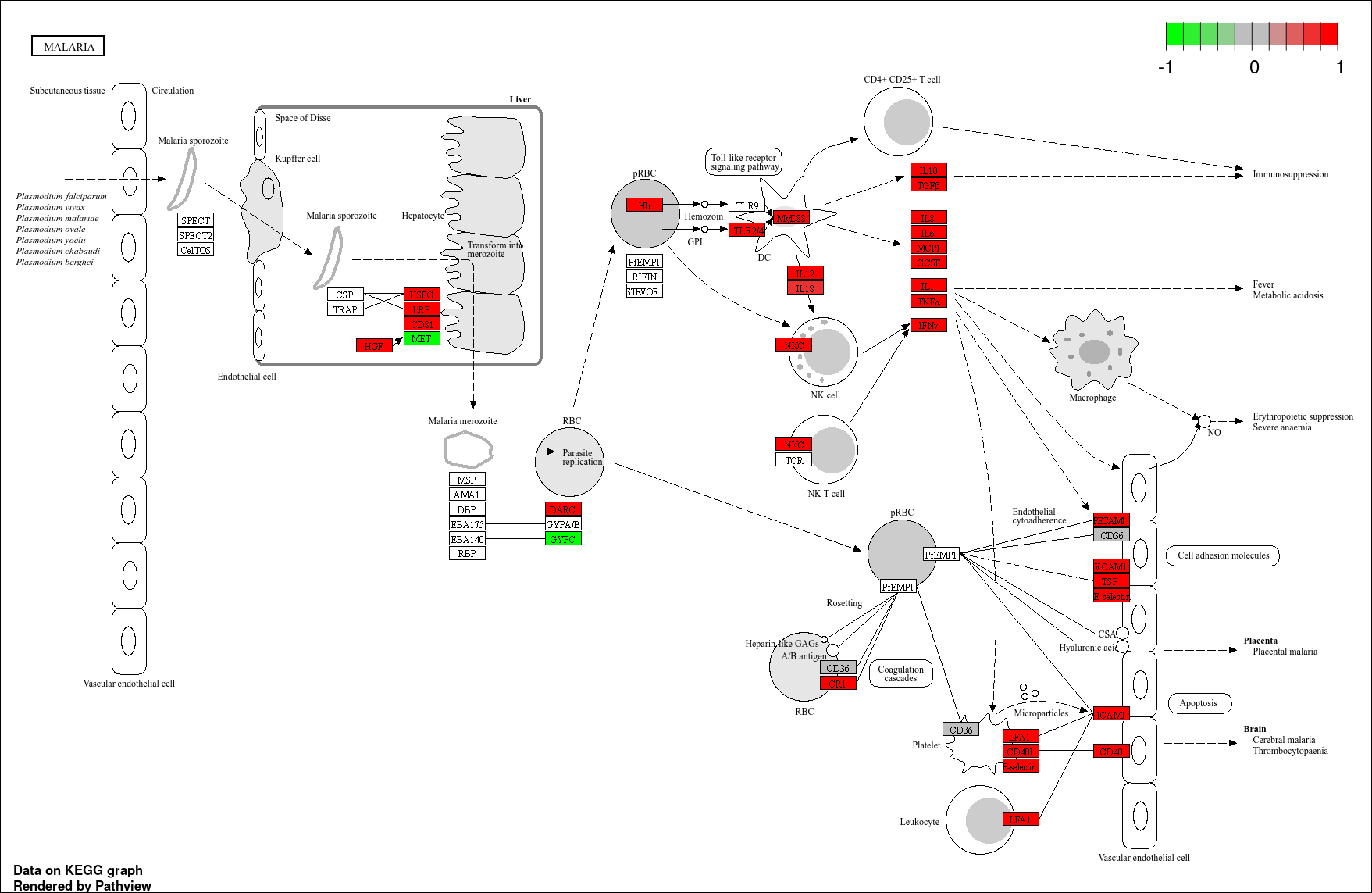


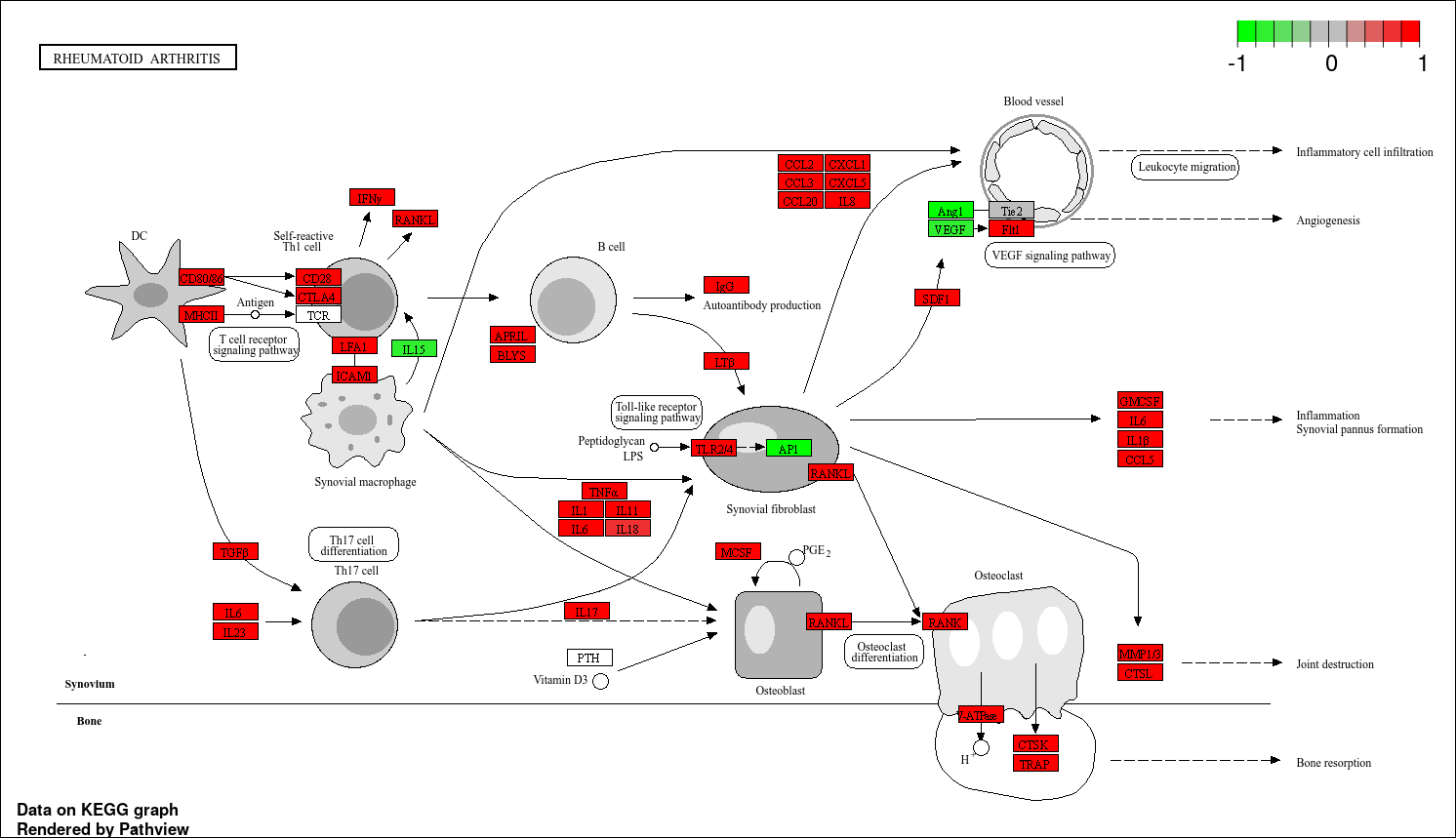


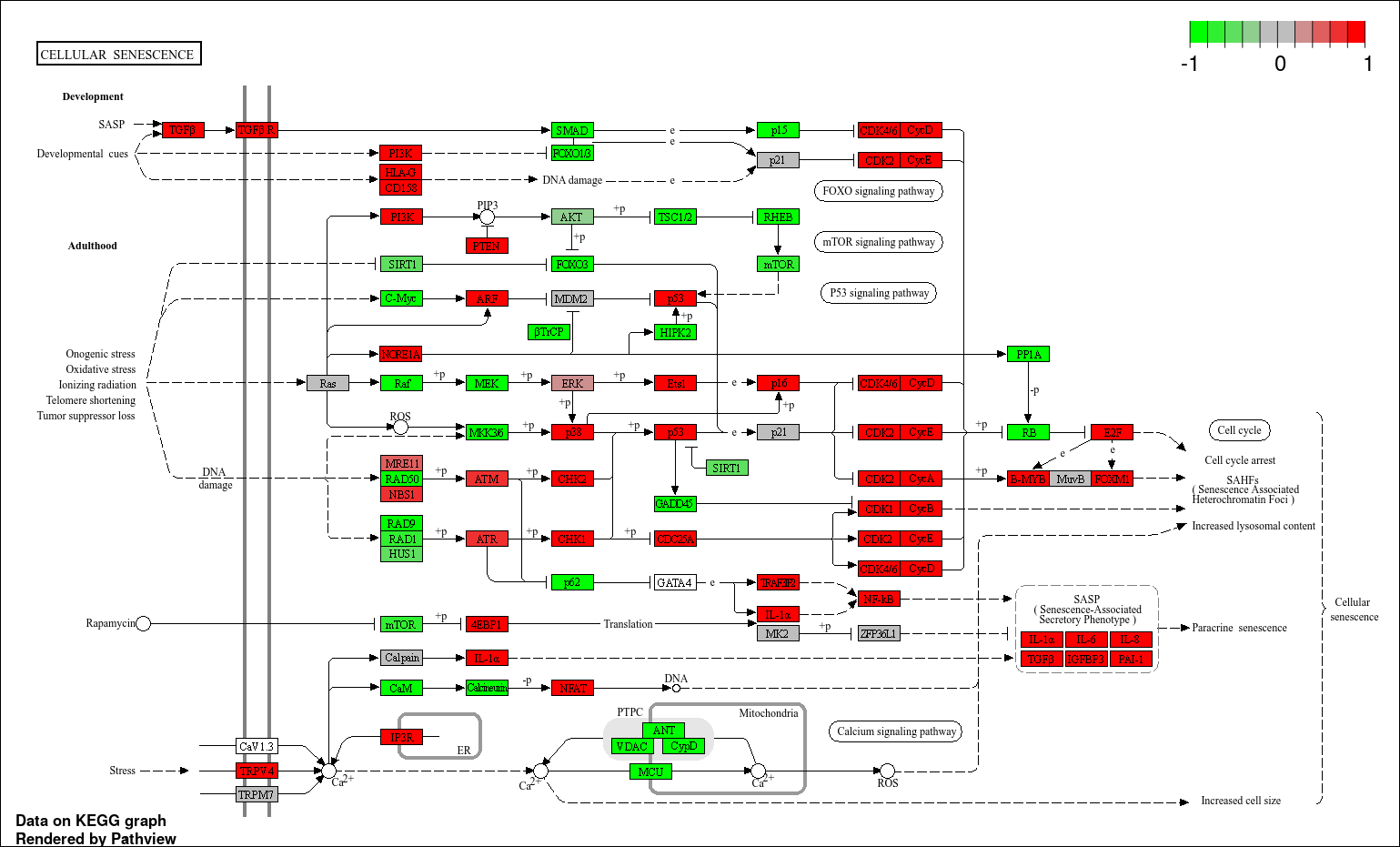


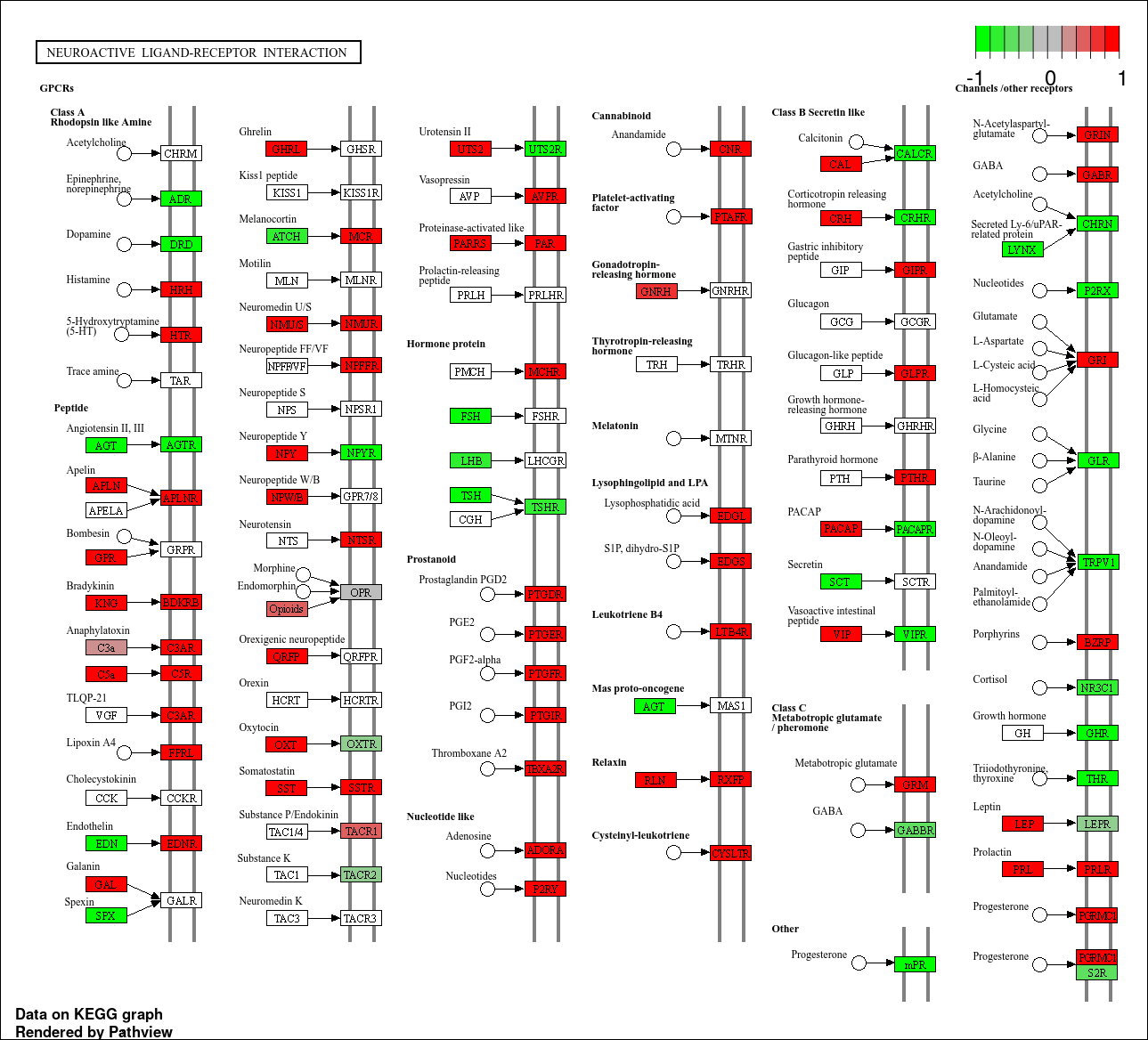


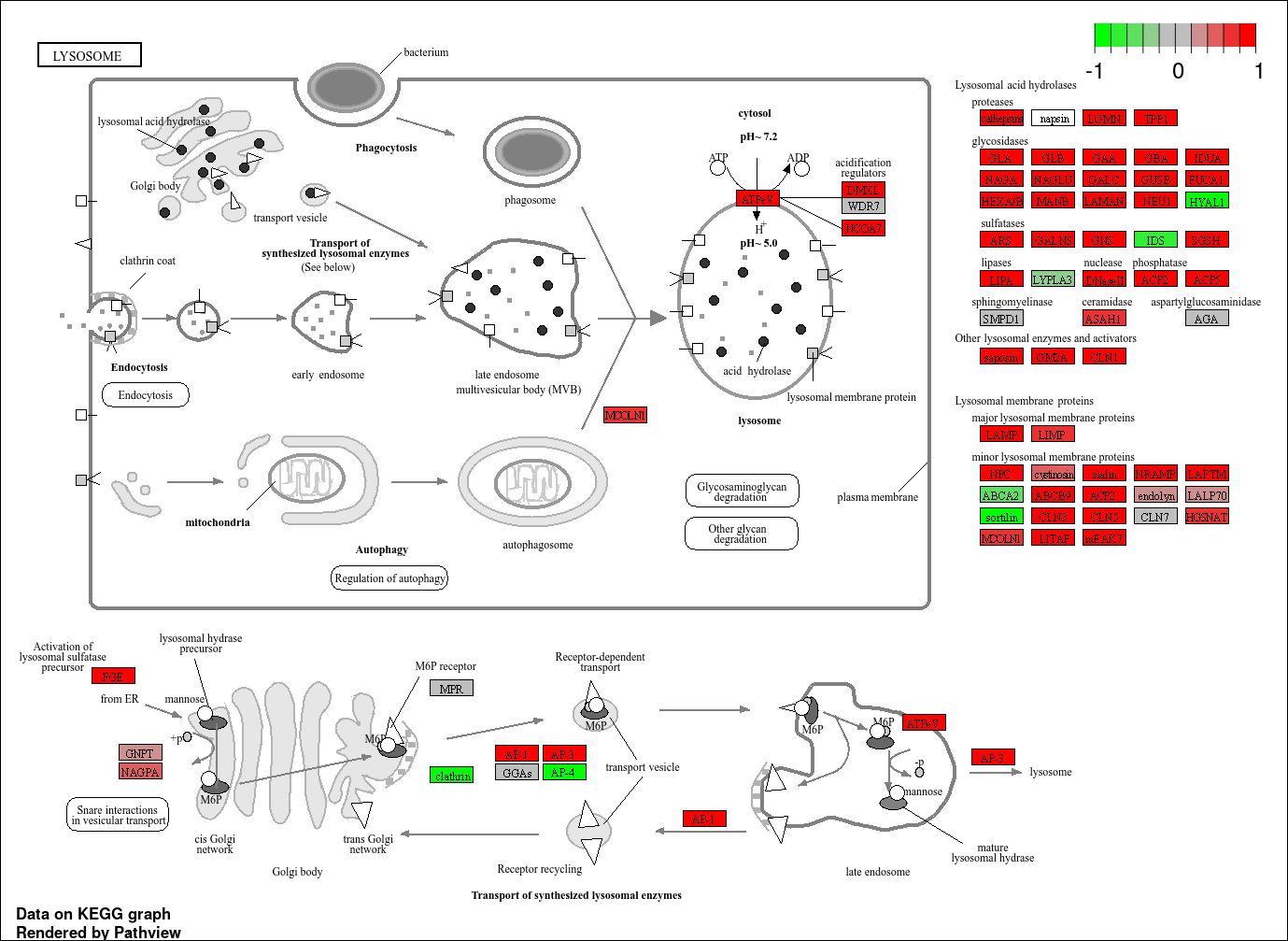


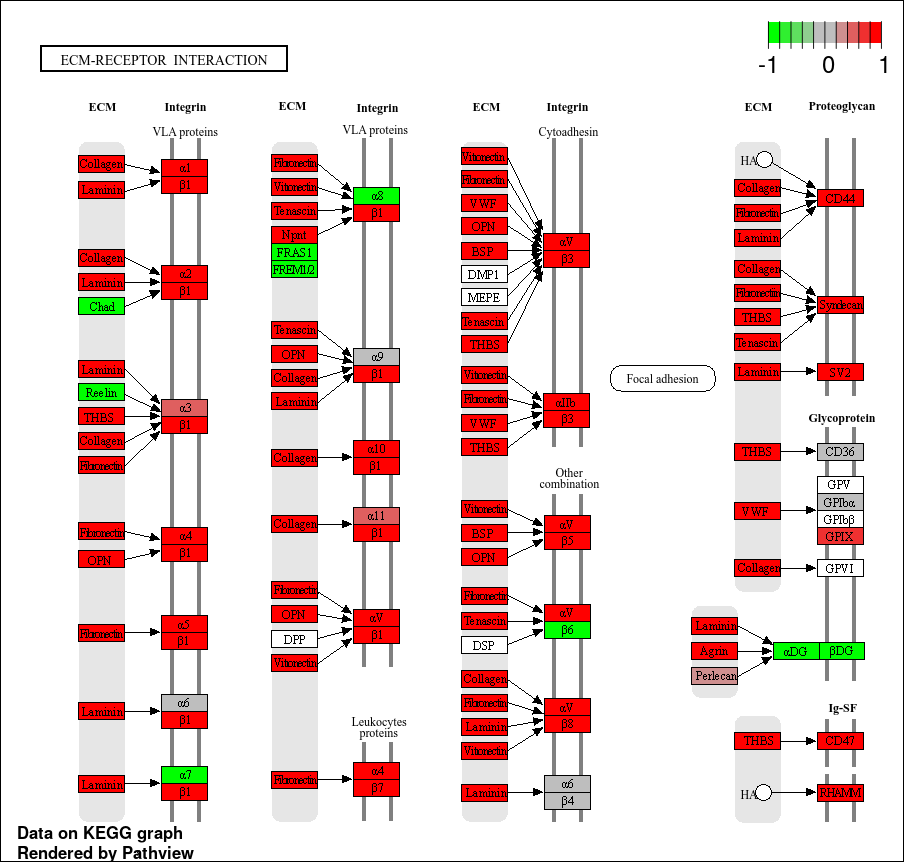


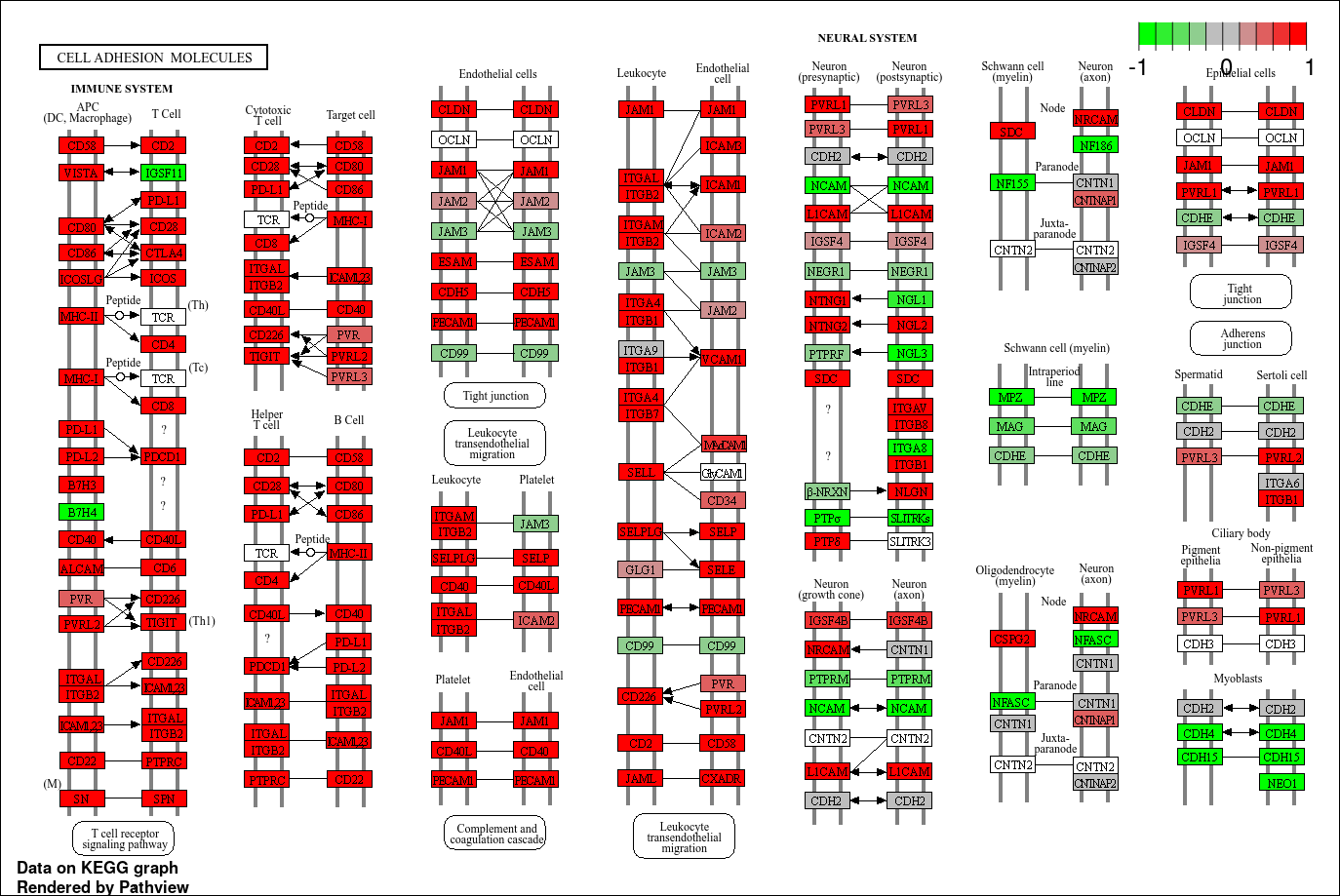


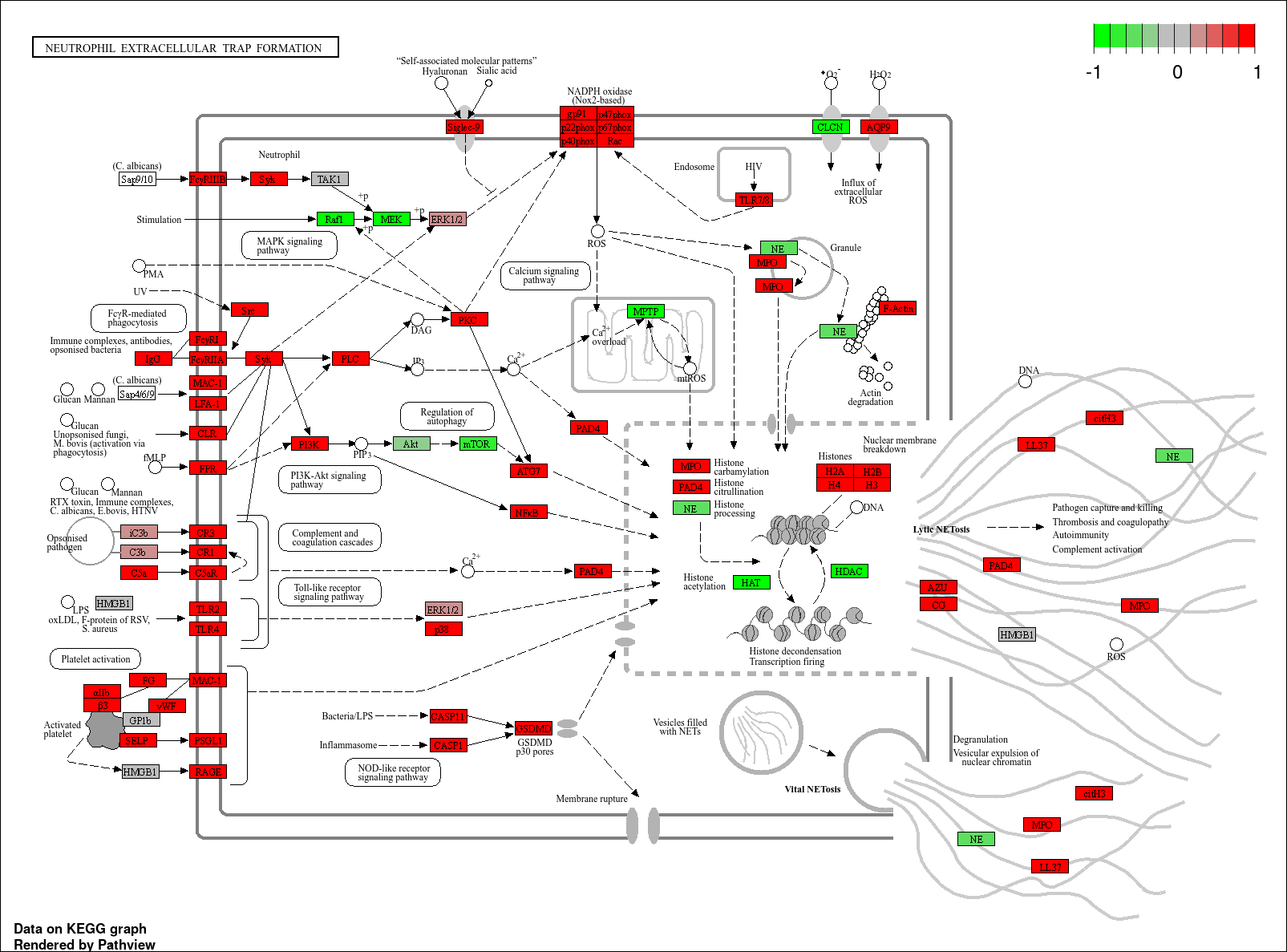


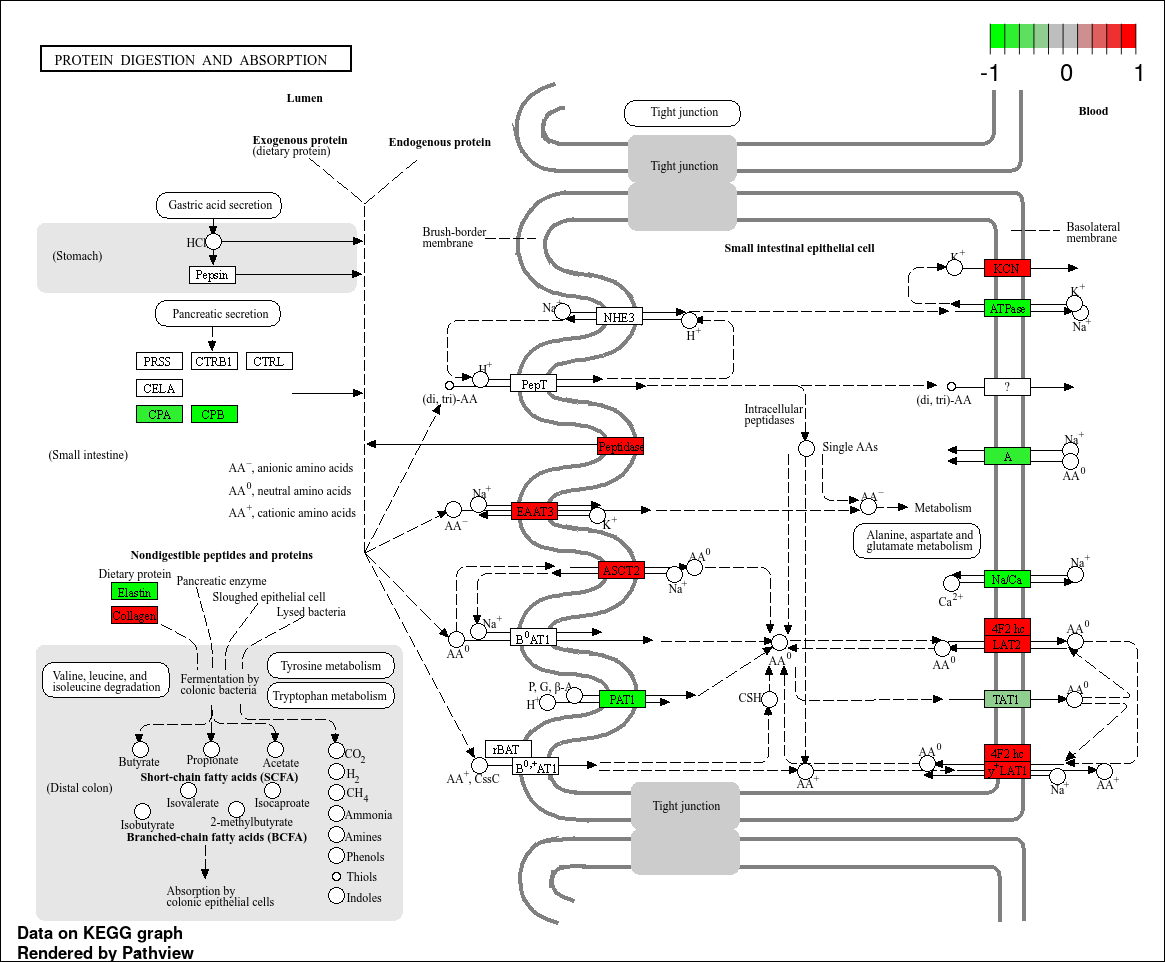


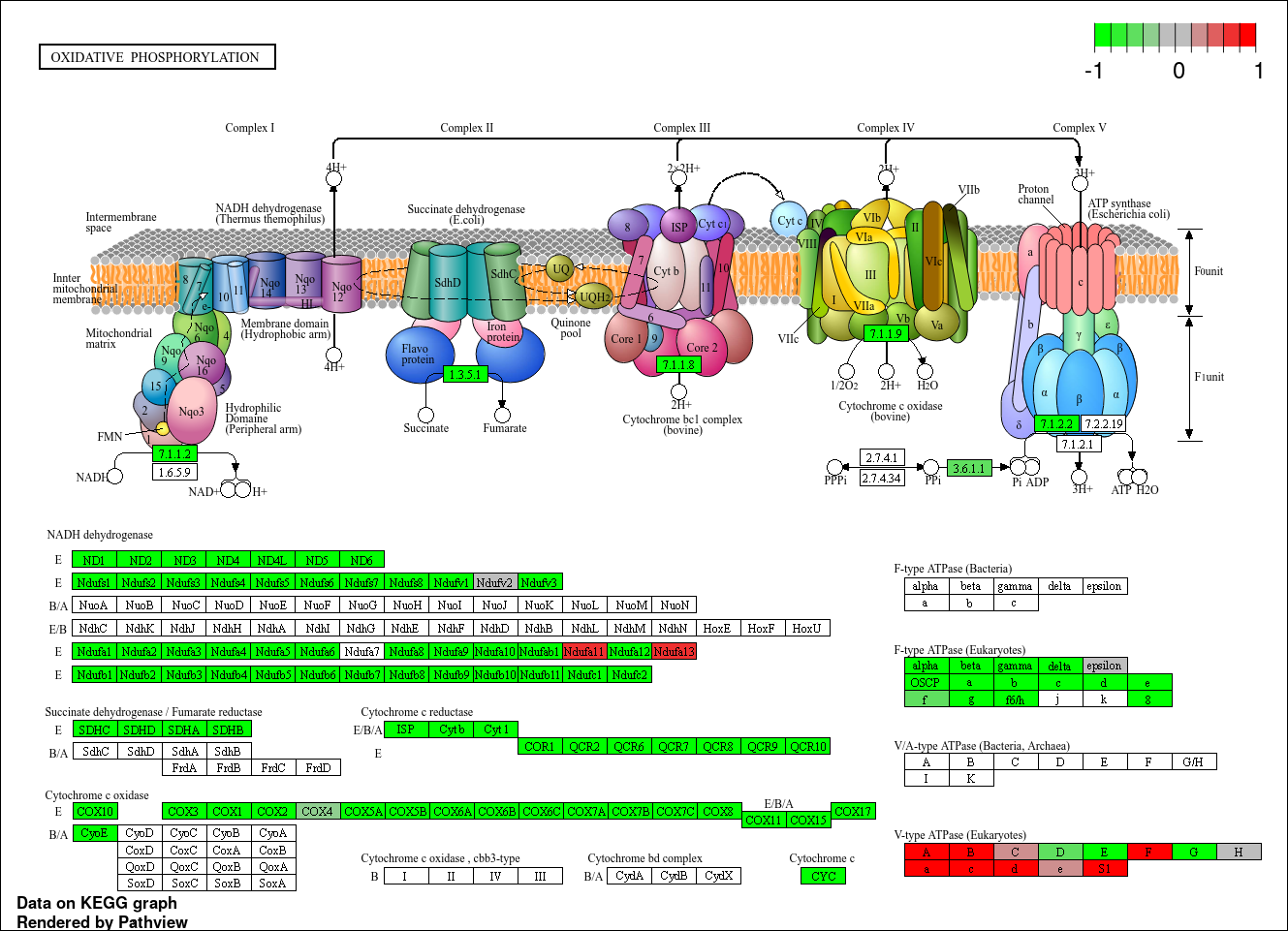


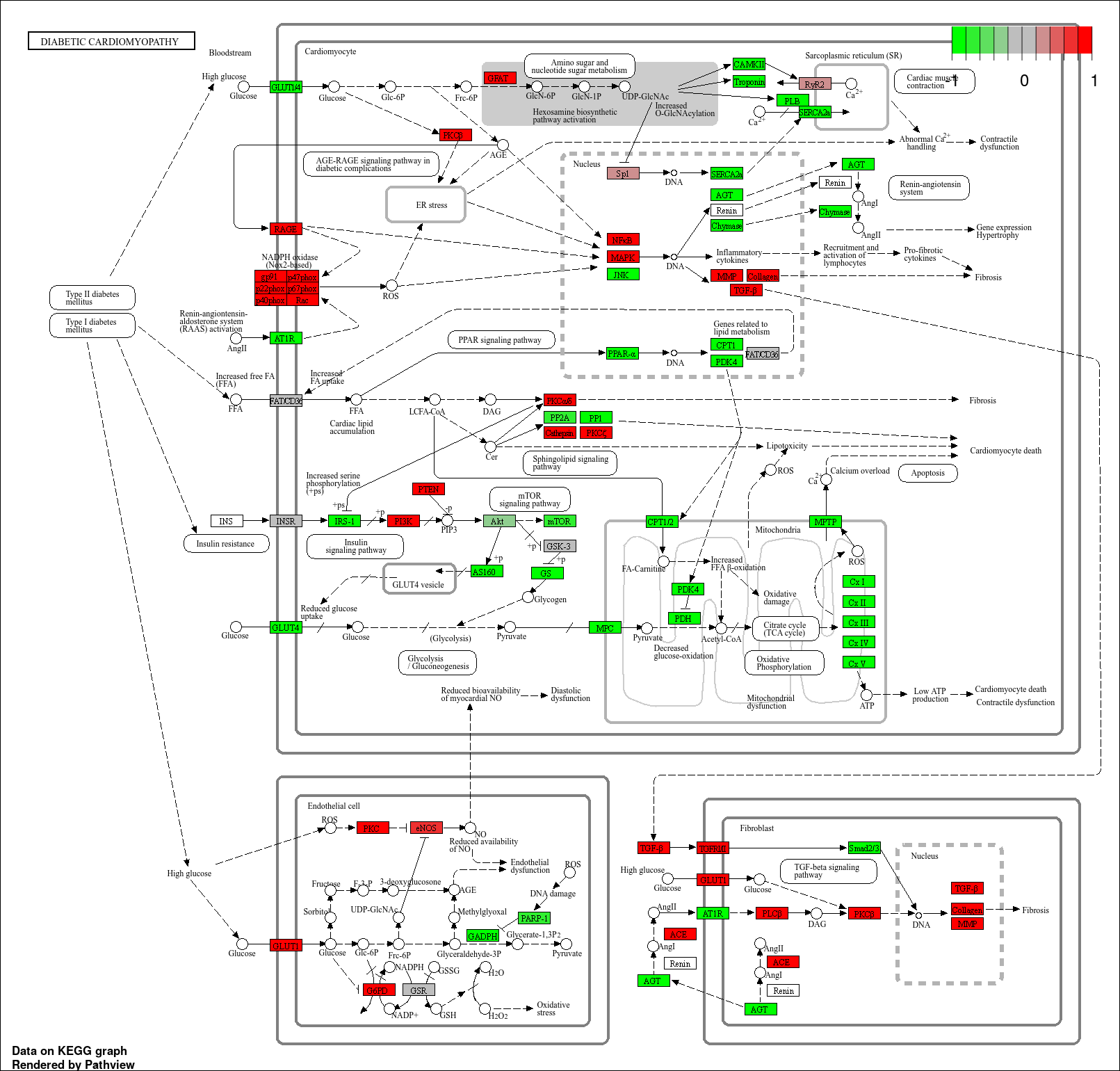


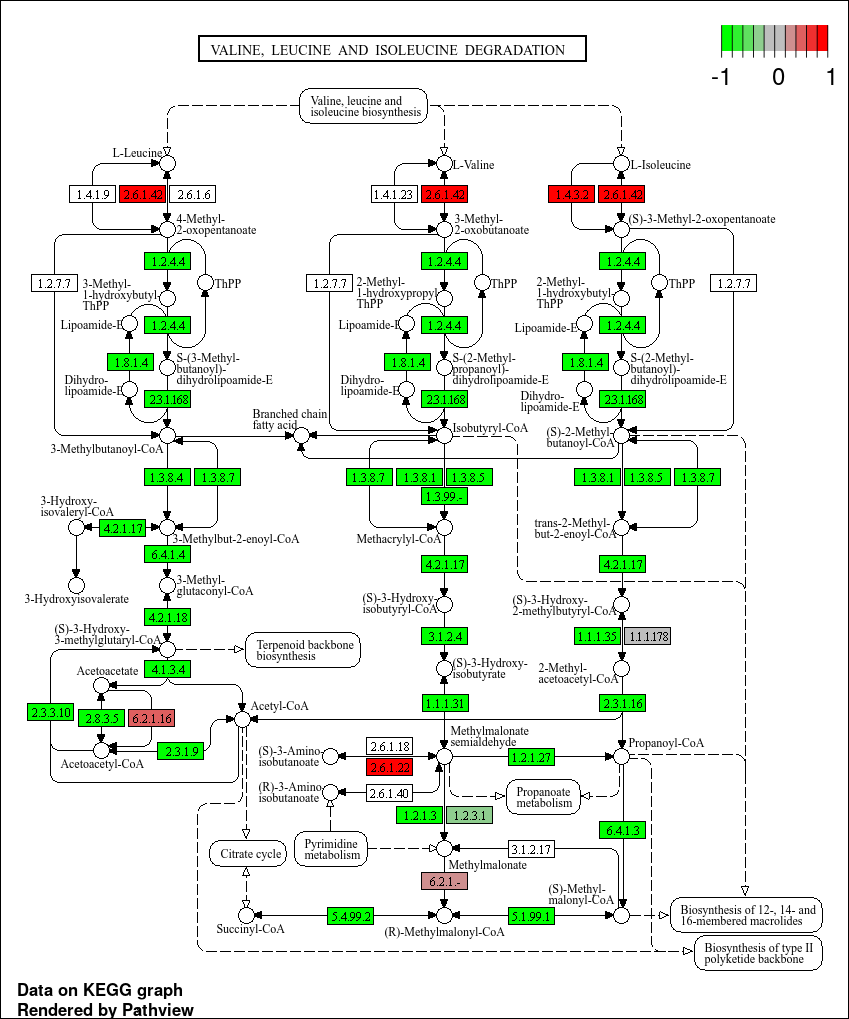


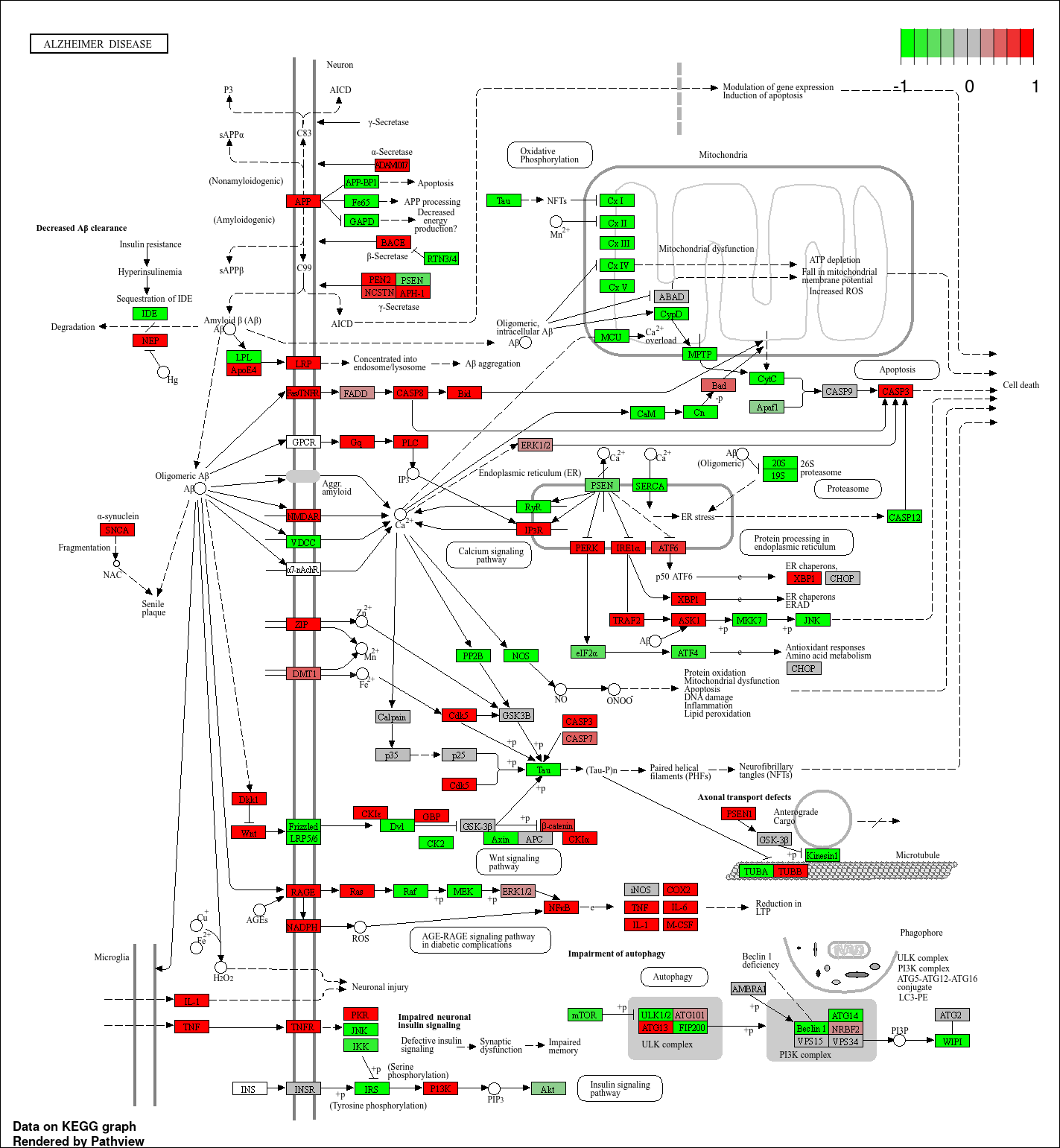


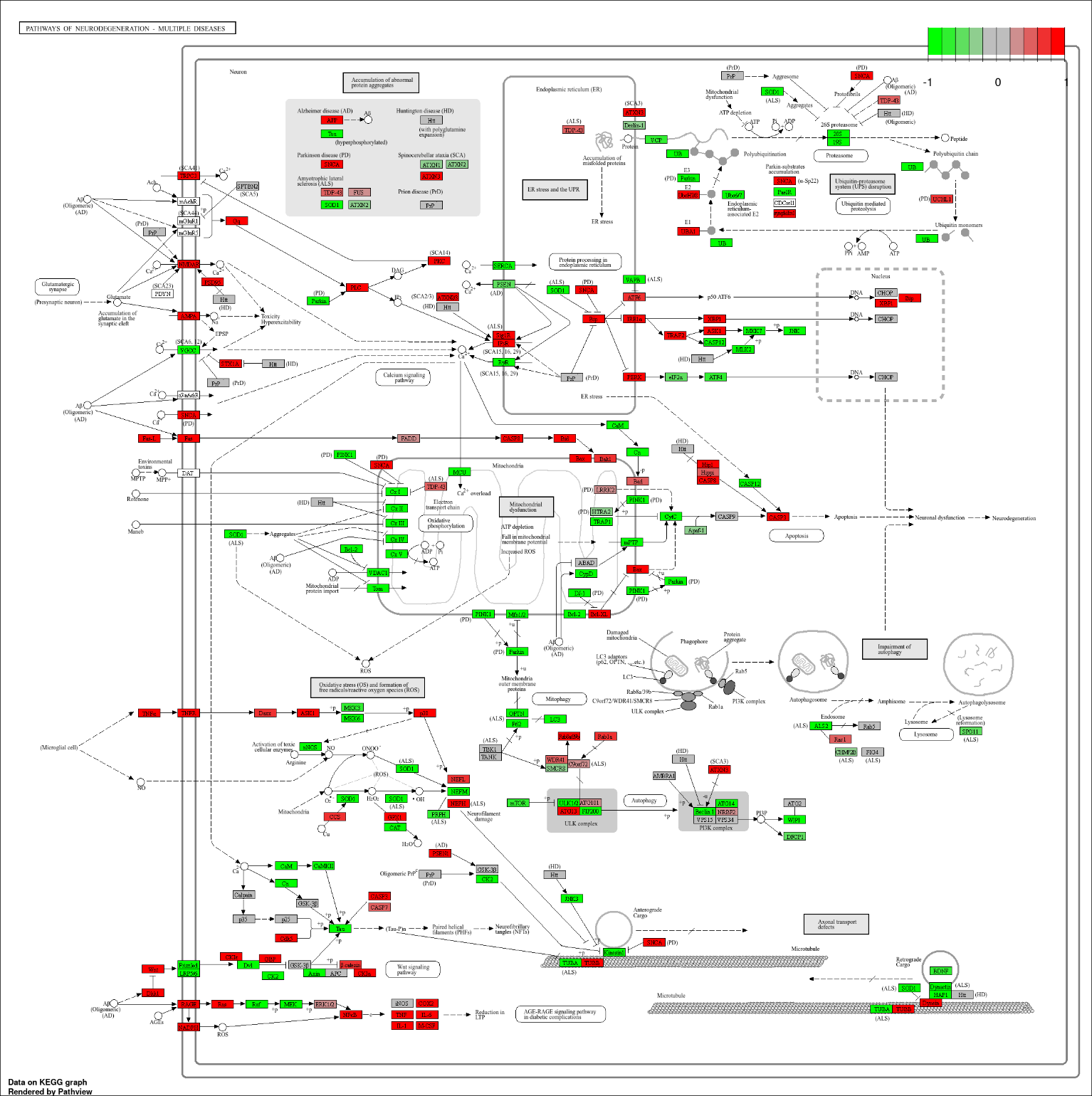


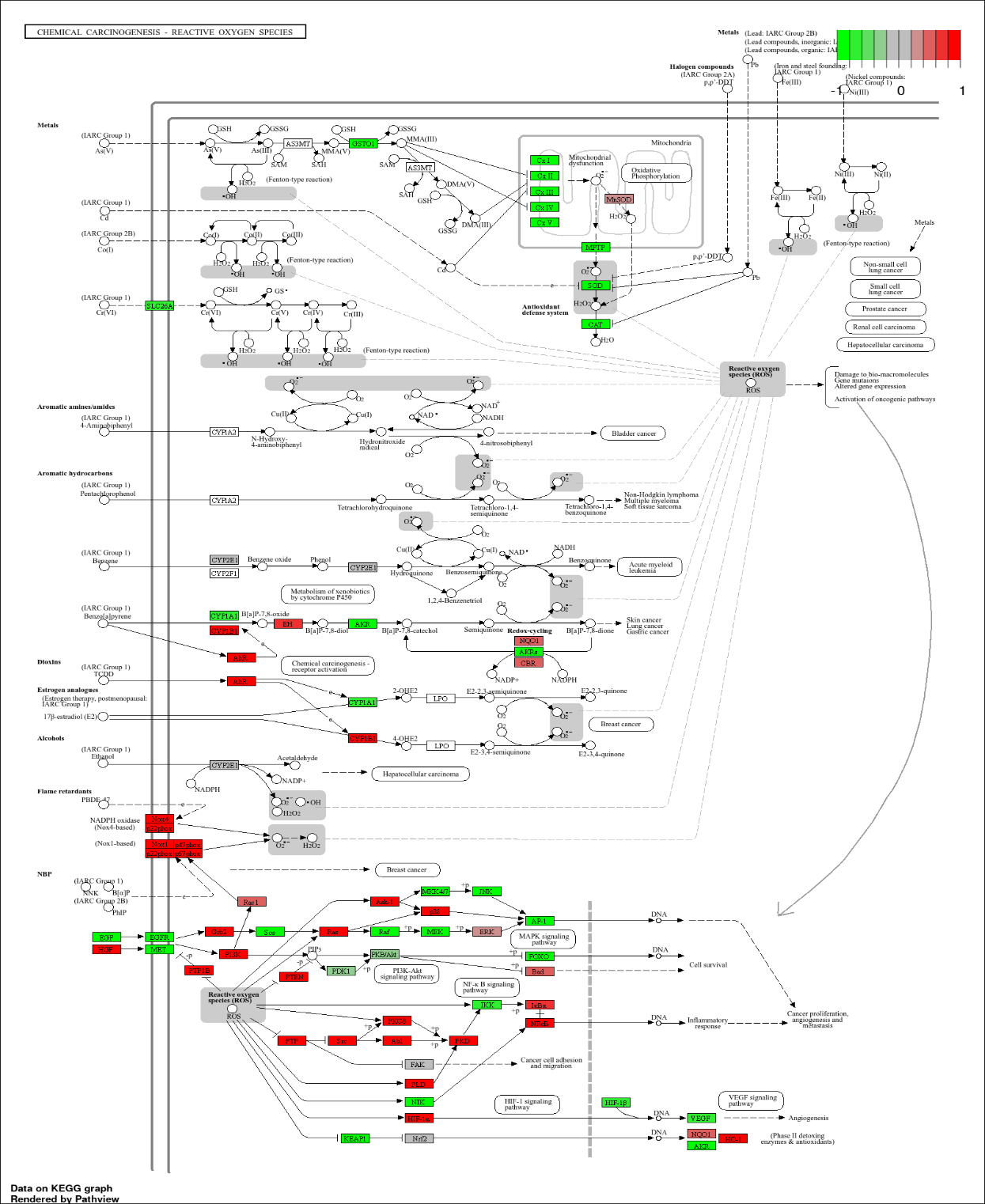


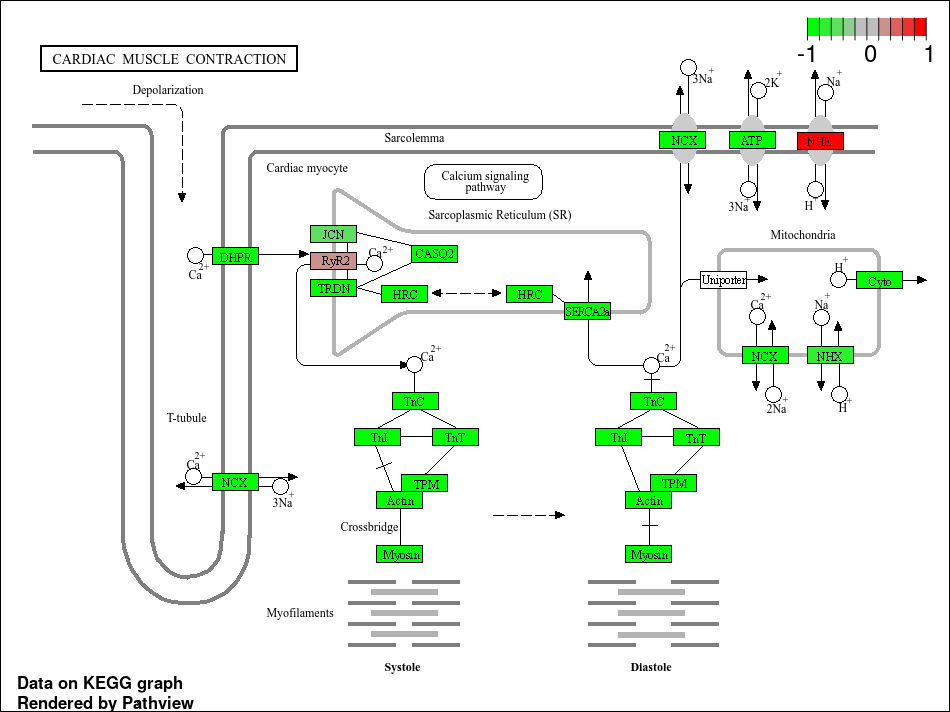


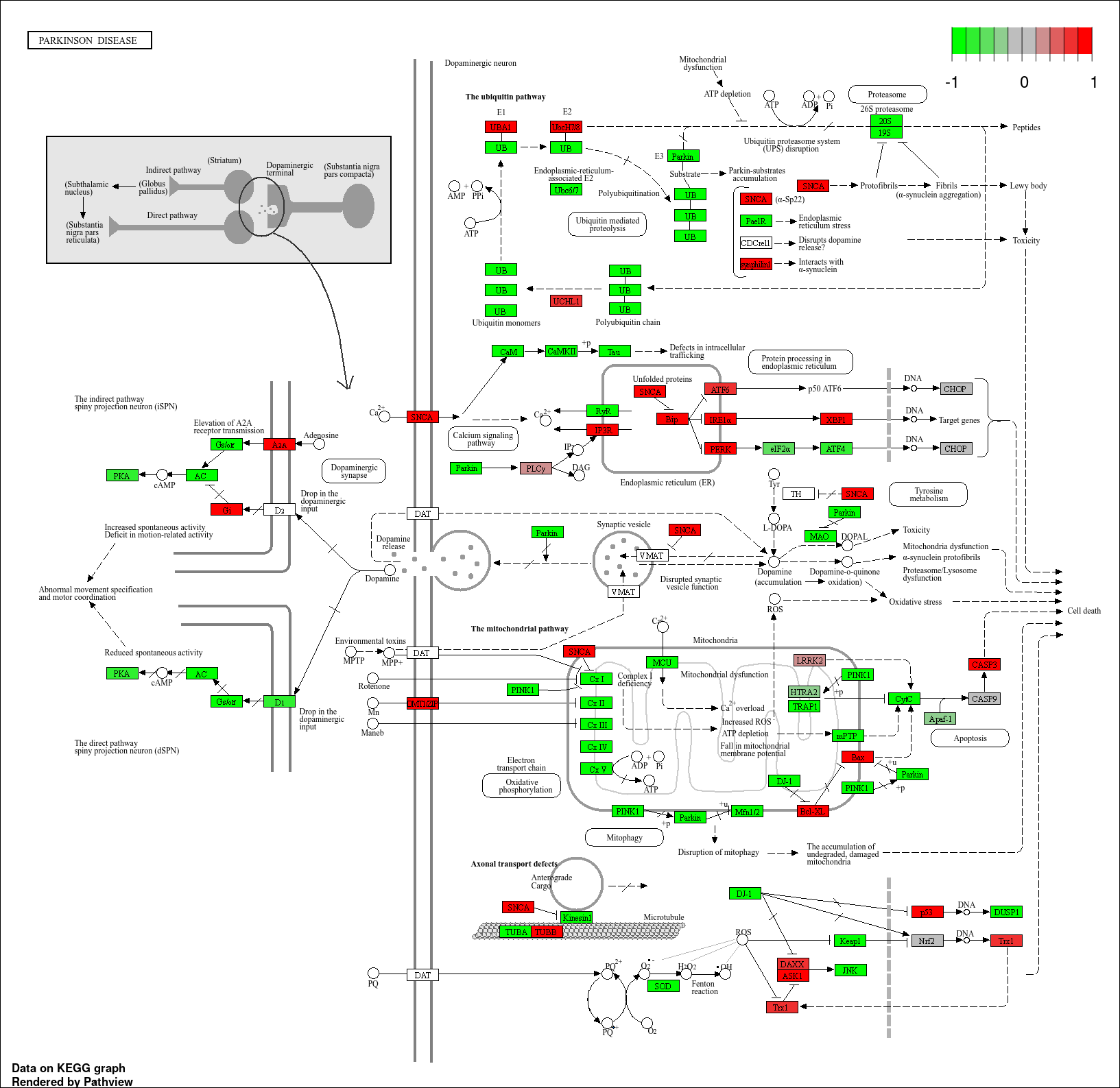


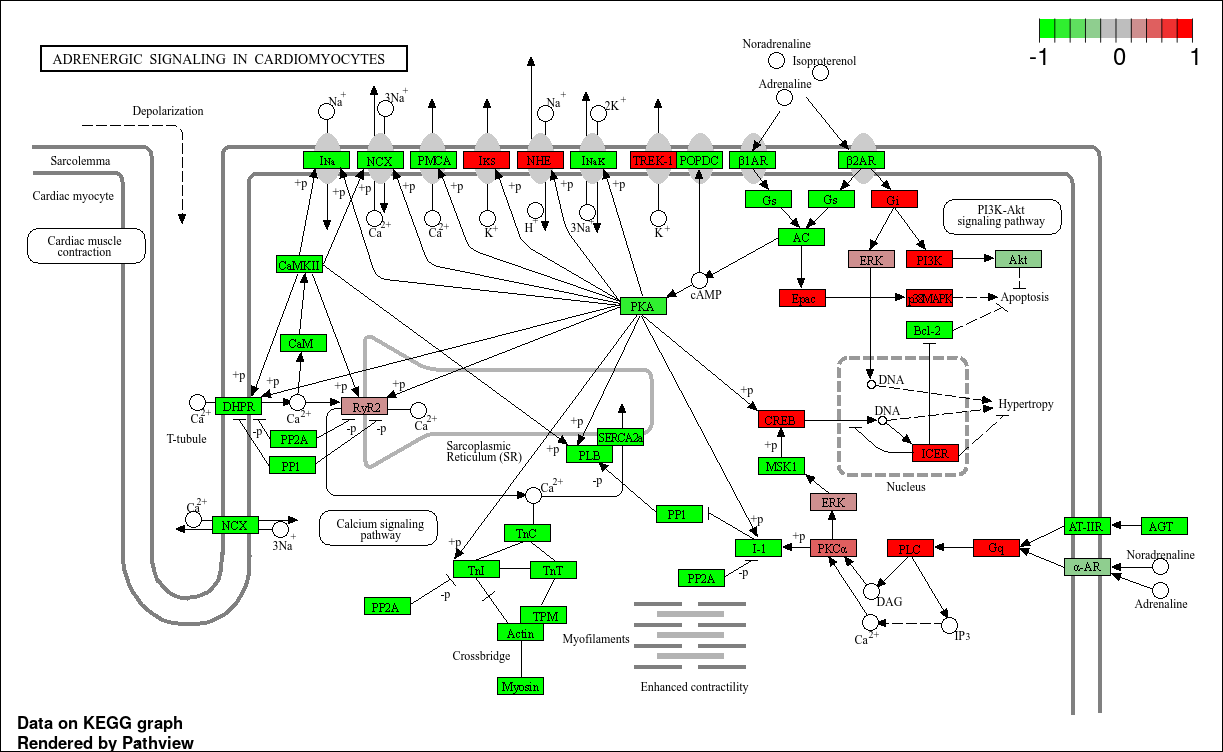


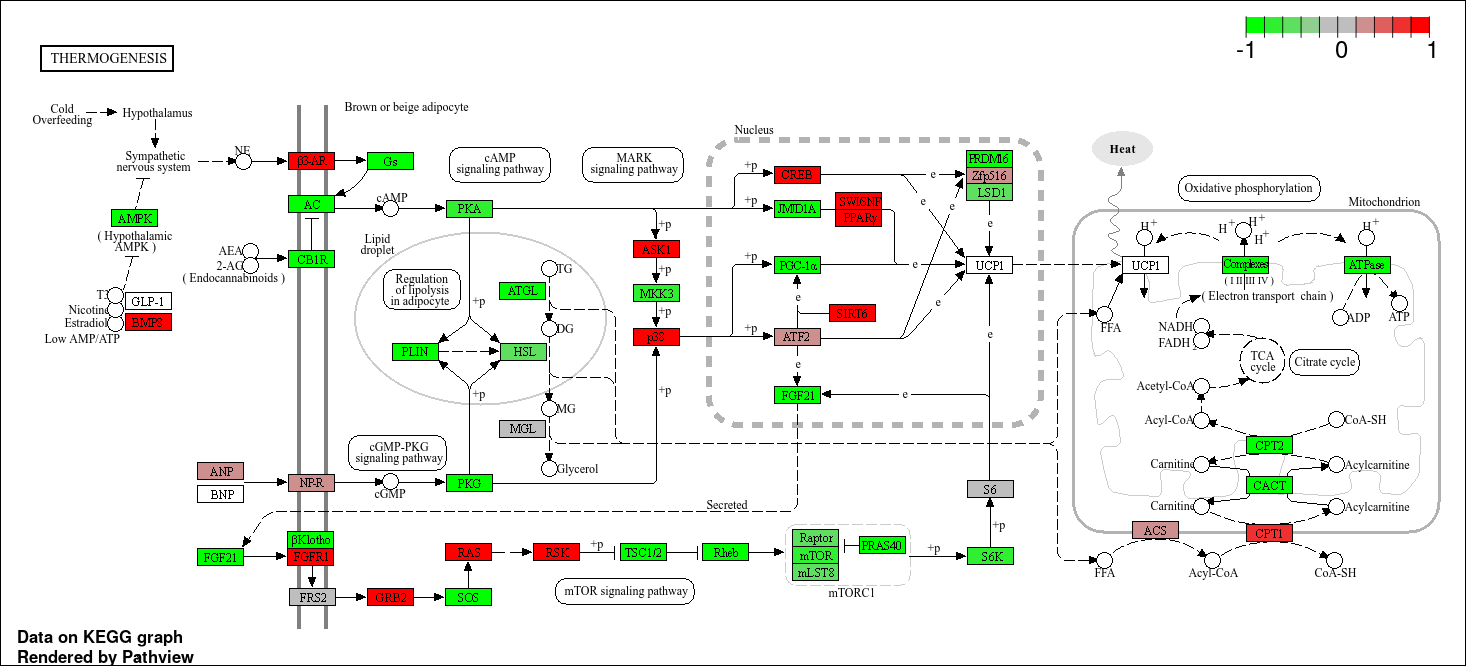

Supplement: Supplementary file 3 [file DataSheet3.docx]
