# Supplementary material for: Comprehensive transcriptomic profiling reveals tissue-specific molecular signatures and dysregulated pathways in human diabetic foot ulcers
Source: Front Endocrinol (Lausanne). 2025 Nov 3;16:1669205. doi: 10.3389/fendo.2025.1669205 (PMC12620202; doi:10.3389/fendo.2025.1669205)
Supplement: Supplementary file 7 [file DataSheet7.docx]

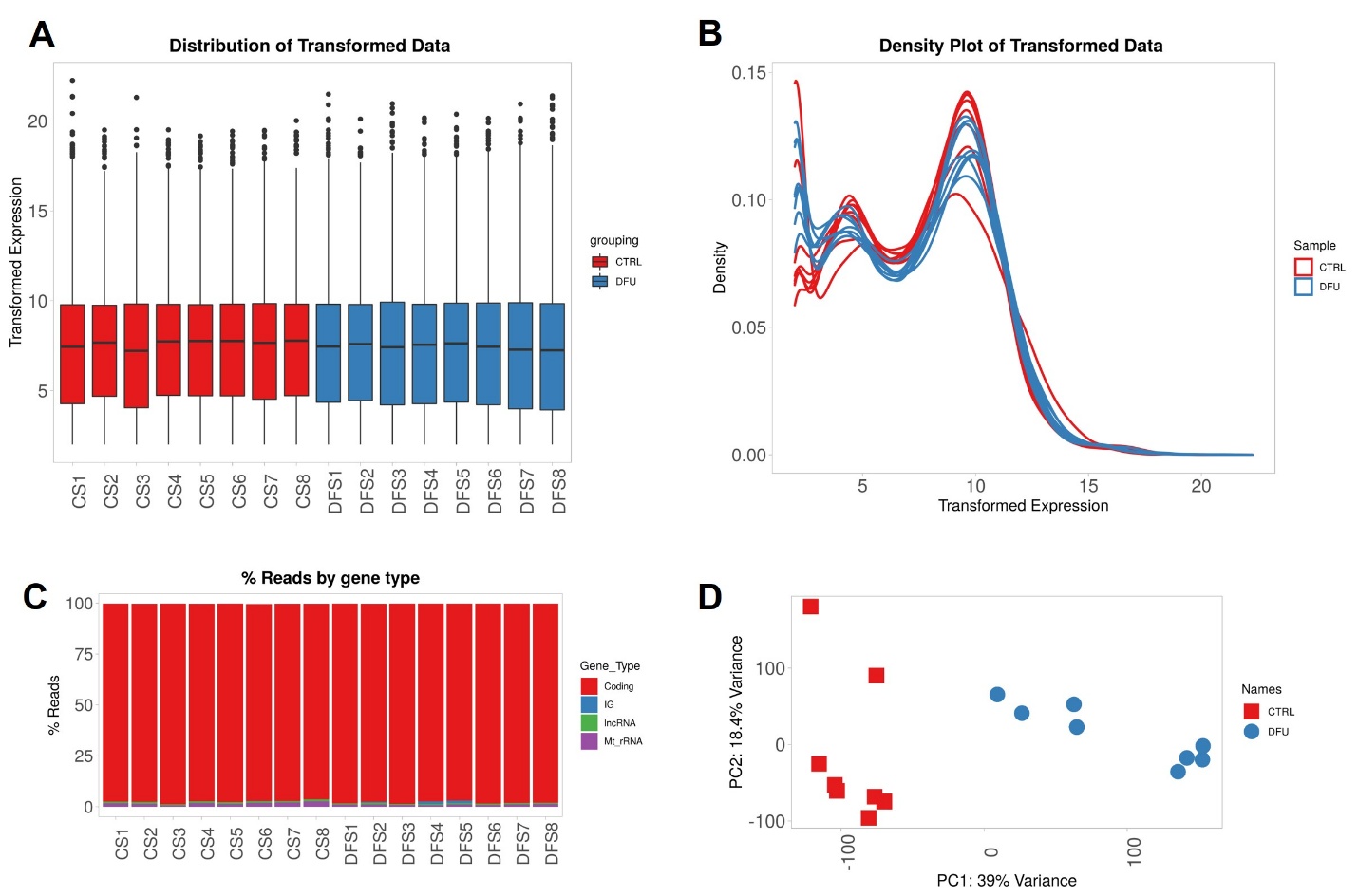


**Figure S1.** **Quality control and exploratory analysis of RNA-seq data from diabetic foot ulcer (DFU) and control (CTRL) in skin samples.** **(A)** Boxplot showing the distribution of transformed gene expression values across all samples after normalization. Red boxes represent control (CS1–CS8) and blue boxes represent DFU samples (DFS1–DFS8), indicating comparable expression distributions across groups. **(B)** Density plot of transformed expression values, between DFU and control samples. **(C)** Bar graph of percentage of sequencing reads categorized by gene type, including coding, immunoglobulin (IG), long non-coding RNA (lncRNA), and mitochondrial rRNA (Mt_rRNA). **(D)** Principal Component Analysis (PCA) plot based on variance-stabilized transformed expression data. Samples cluster distinctly by group, with principal component 1 (PC1) explaining 39% of the variance and PC2 explaining 18.4%, between DFU and CTRL skin samples.


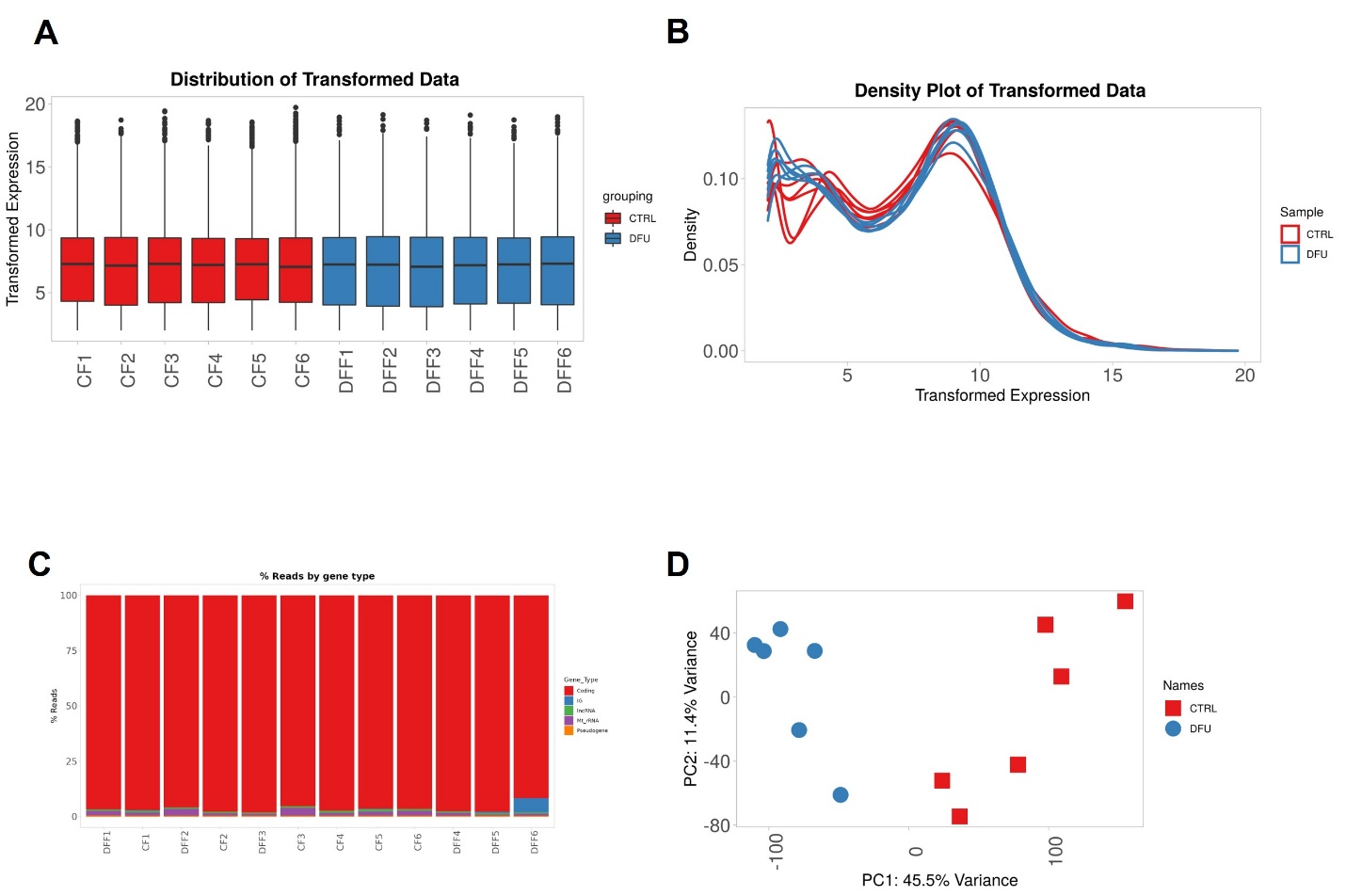


**Figure S2.** **Quality control and exploratory analysis of RNA-seq data from diabetic foot ulcer (DFU) and control (CTRL) in fat samples.** **(A)** Boxplot showing the distribution of transformed gene expression values across all samples after normalization. Red boxes represent control (CF1–CF6) and blue boxes represent DFU samples (DFF1–DFF6), indicating comparable expression distributions across groups. **(B)** Density plot of transformed expression values, between DFU and control samples. **(C)** Bar graph of percentage of sequencing reads categorized by gene type, including coding, immunoglobulin (IG), long non-coding RNA (lncRNA), and mitochondrial rRNA (Mt_rRNA). **(D)** Principal Component Analysis (PCA) plot based on variance-stabilized transformed expression data. Samples cluster distinctly by group, with principal component 1 (PC1) explaining 45.5% of the variance and PC2 explaining 11.4%, between DFU and CTRL fat samples.


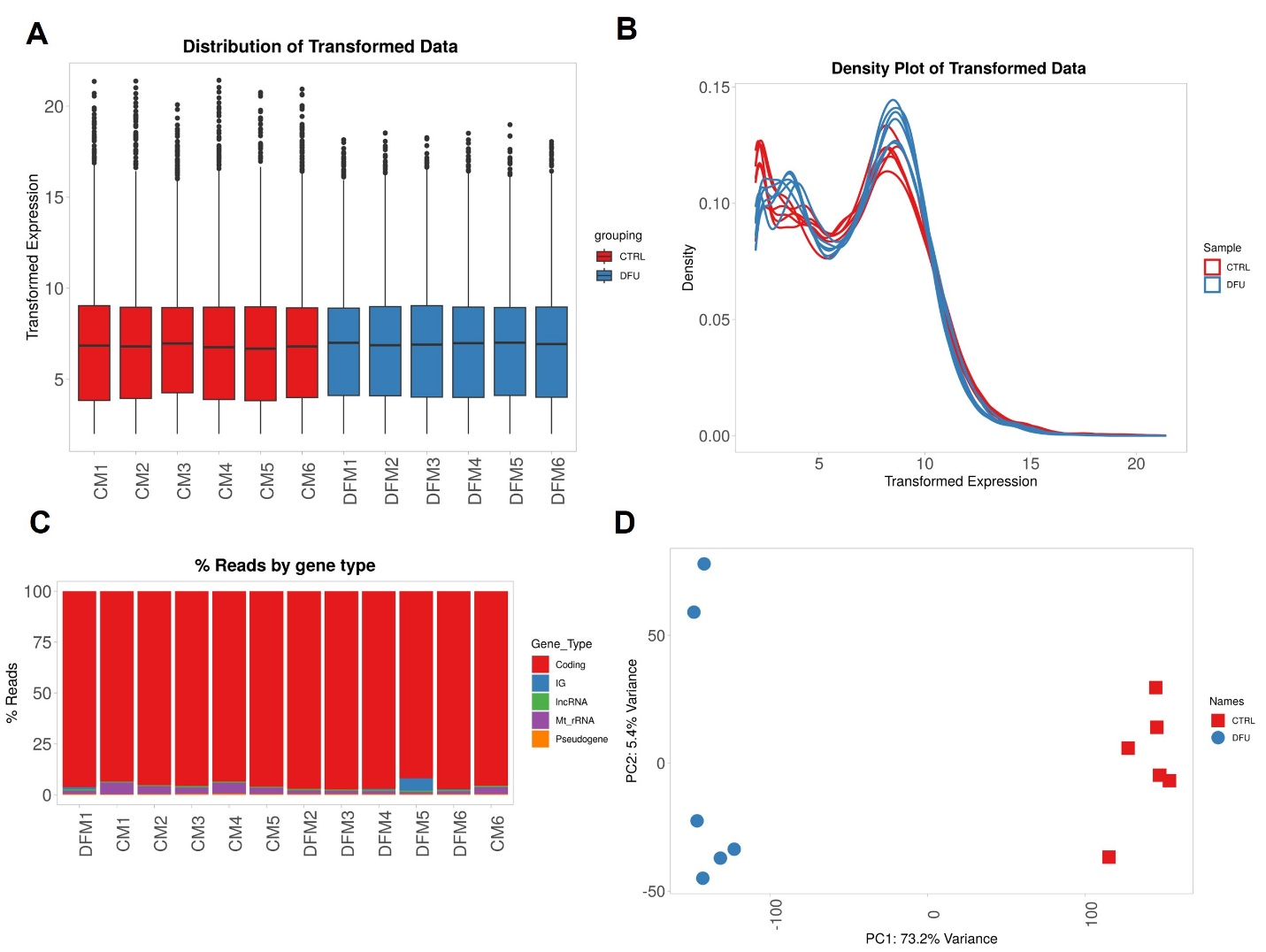


**Figure S3. Quality control and exploratory analysis of RNA-seq data from diabetic foot ulcer (DFU) and control (CTRL) in muscles samples.** **(A)** Boxplot showing the distribution of transformed gene expression values across all samples after normalization. Red boxes represent control (CM1–CM6) and blue boxes represent DFU samples (DFM1–DFM6), indicating comparable expression distributions across groups. **(B)** Density plot of transformed expression values, between DFU and control samples. **(C)** Bar graph of percentage of sequencing reads categorized by gene type, including coding, immunoglobulin (IG), long non-coding RNA (lncRNA), and mitochondrial rRNA (Mt_rRNA). **(D)** Principal Component Analysis (PCA) plot based on variance-stabilized transformed expression data. Samples cluster distinctly by group, with principal component 1 (PC1) explaining 73.2% of the variance and PC2 explaining 5.4%, between DFU and CTRL muscle samples.


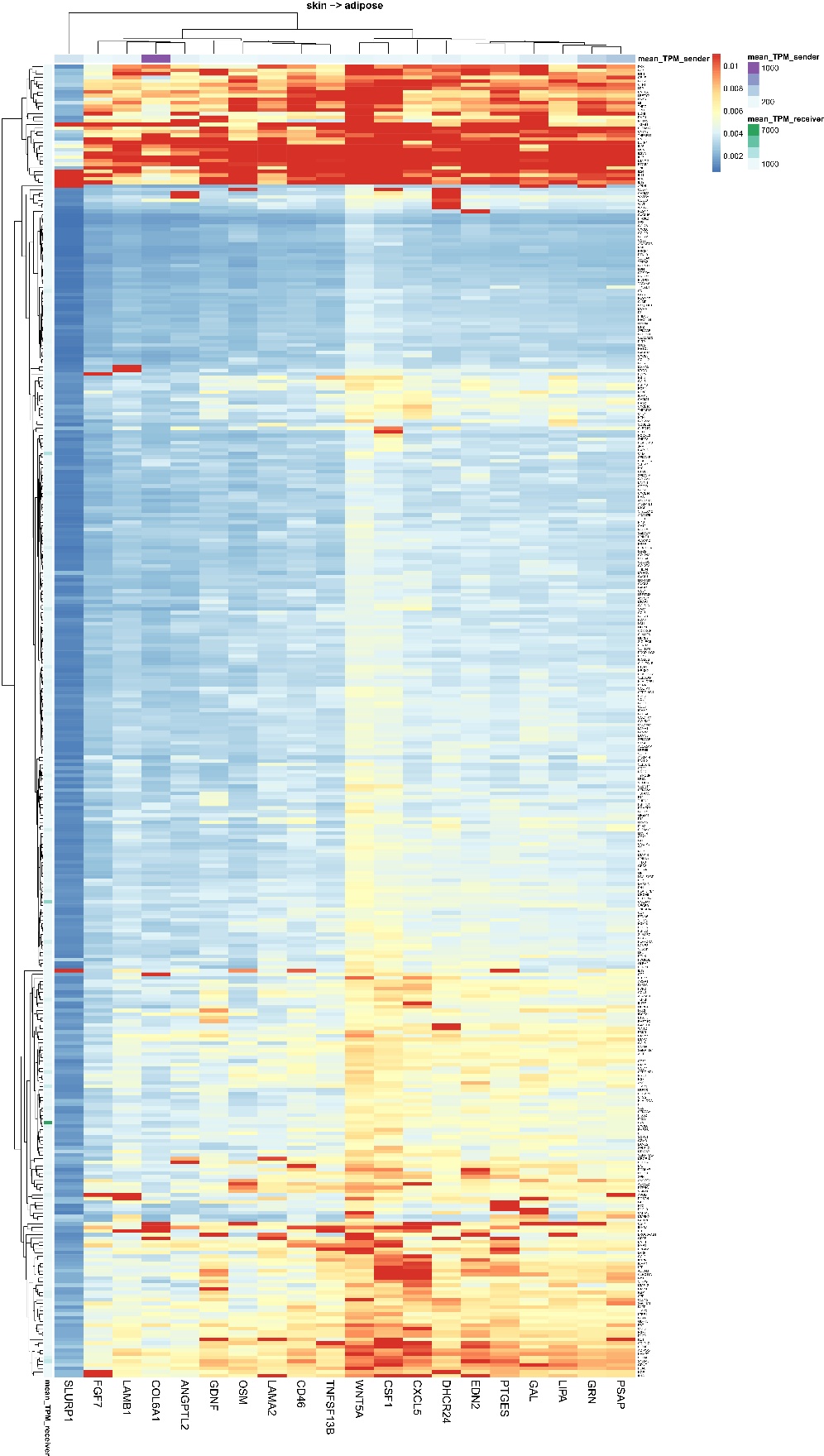


**Figure S4. Skin to adipose paracrine signaling.**

Heatmap of ligand–receptor expression pairs highlighting skin-derived signals targeting adipose receptors.


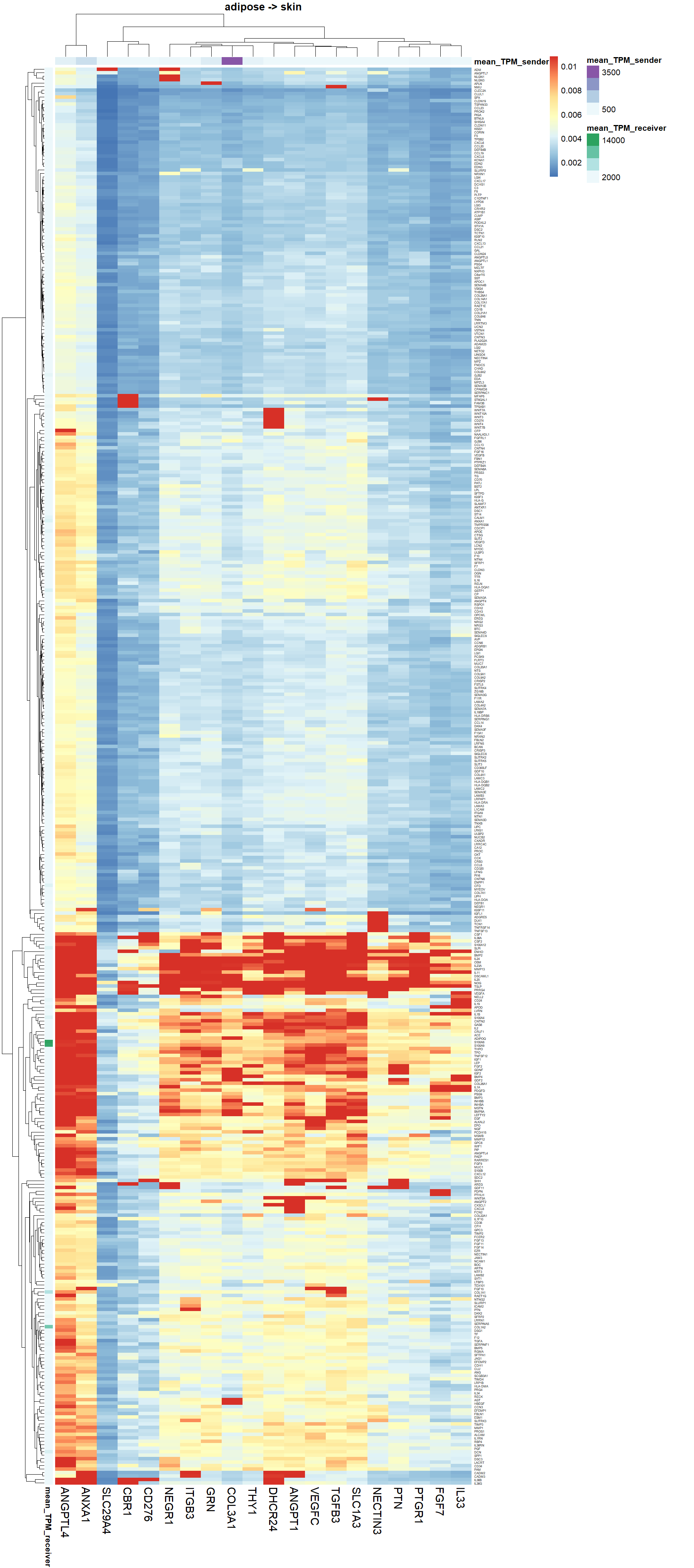


**Figure S5. Adipose to skin paracrine signaling.**

Heatmap of ligand–receptor expression pairs highlighting adipose-derived signals targeting skin receptors.


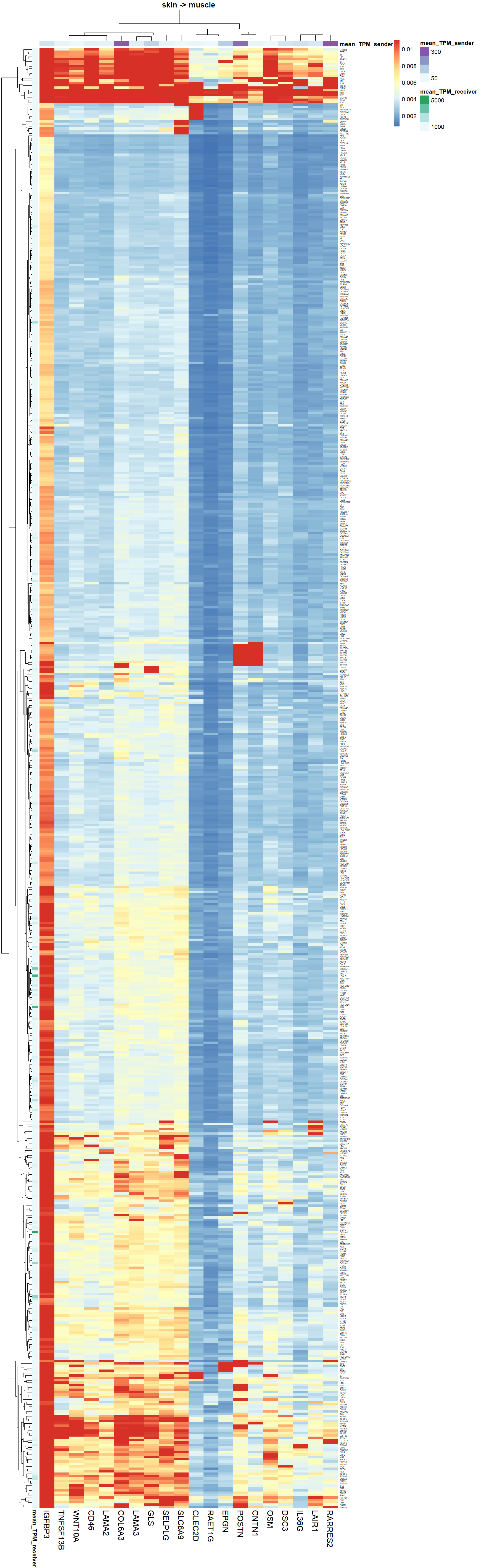


**Figure S6. Skin to muscle paracrine signaling.**

Heatmap of ligand–receptor expression pairs highlighting skin-derived signals targeting muscle receptors.


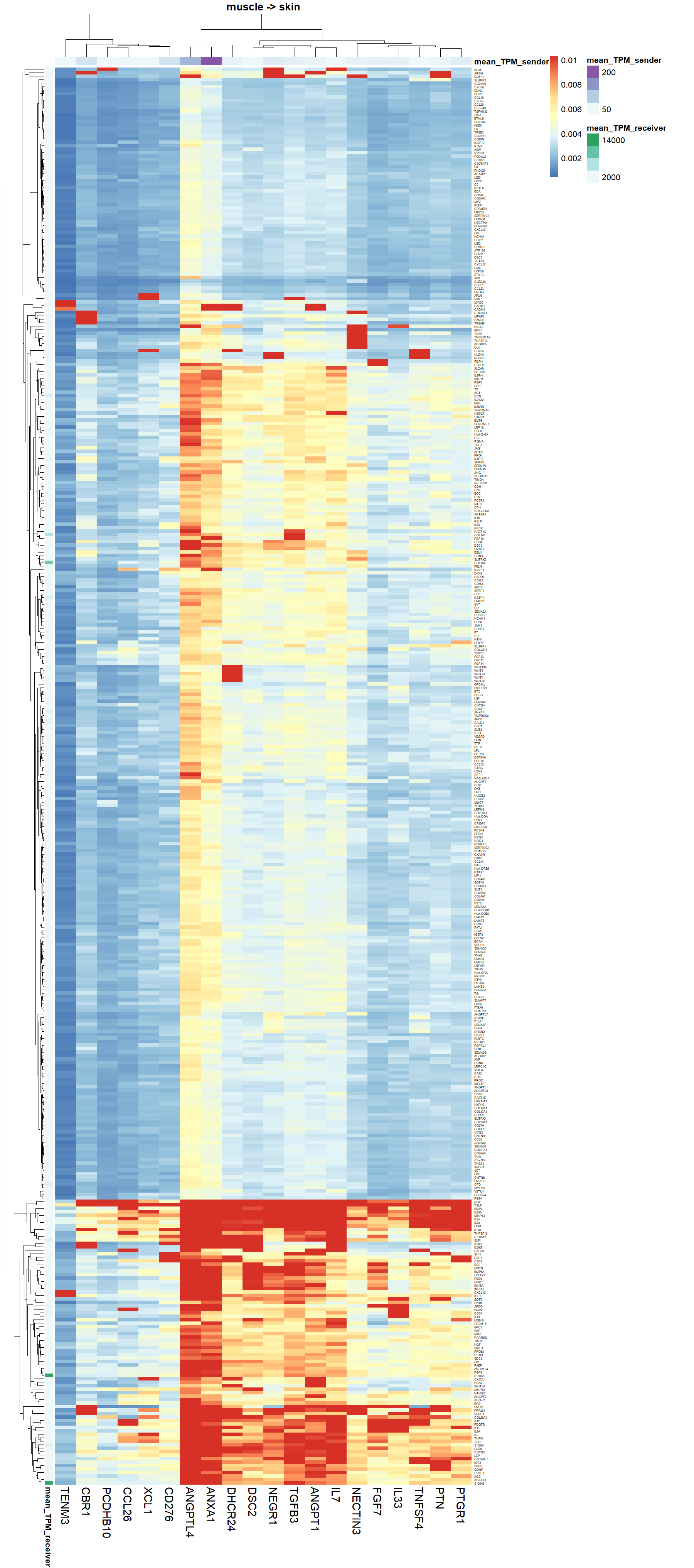


**Figure S7. Muscle to skin paracrine signaling.**

Heatmap of ligand–receptor expression pairs highlighting muscle-derived signals targeting skin receptors.


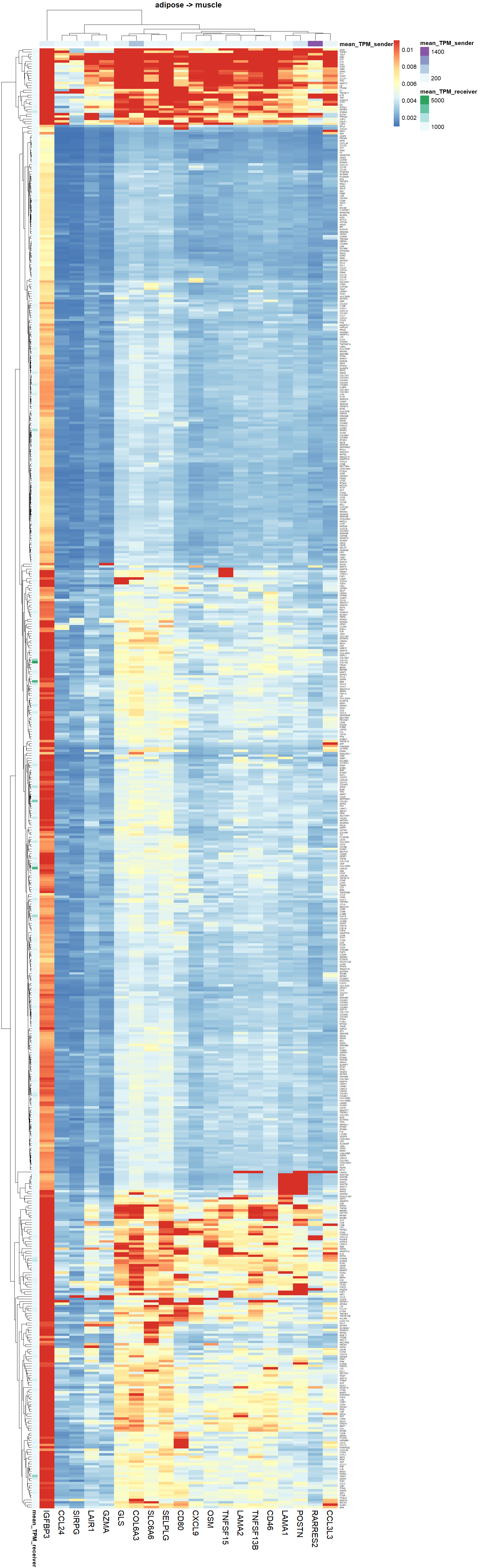


**Figure S8. Adipose to muscle paracrine signaling.**

Heatmap of ligand–receptor expression pairs highlighting adipose-derived signals targeting muscle receptors.


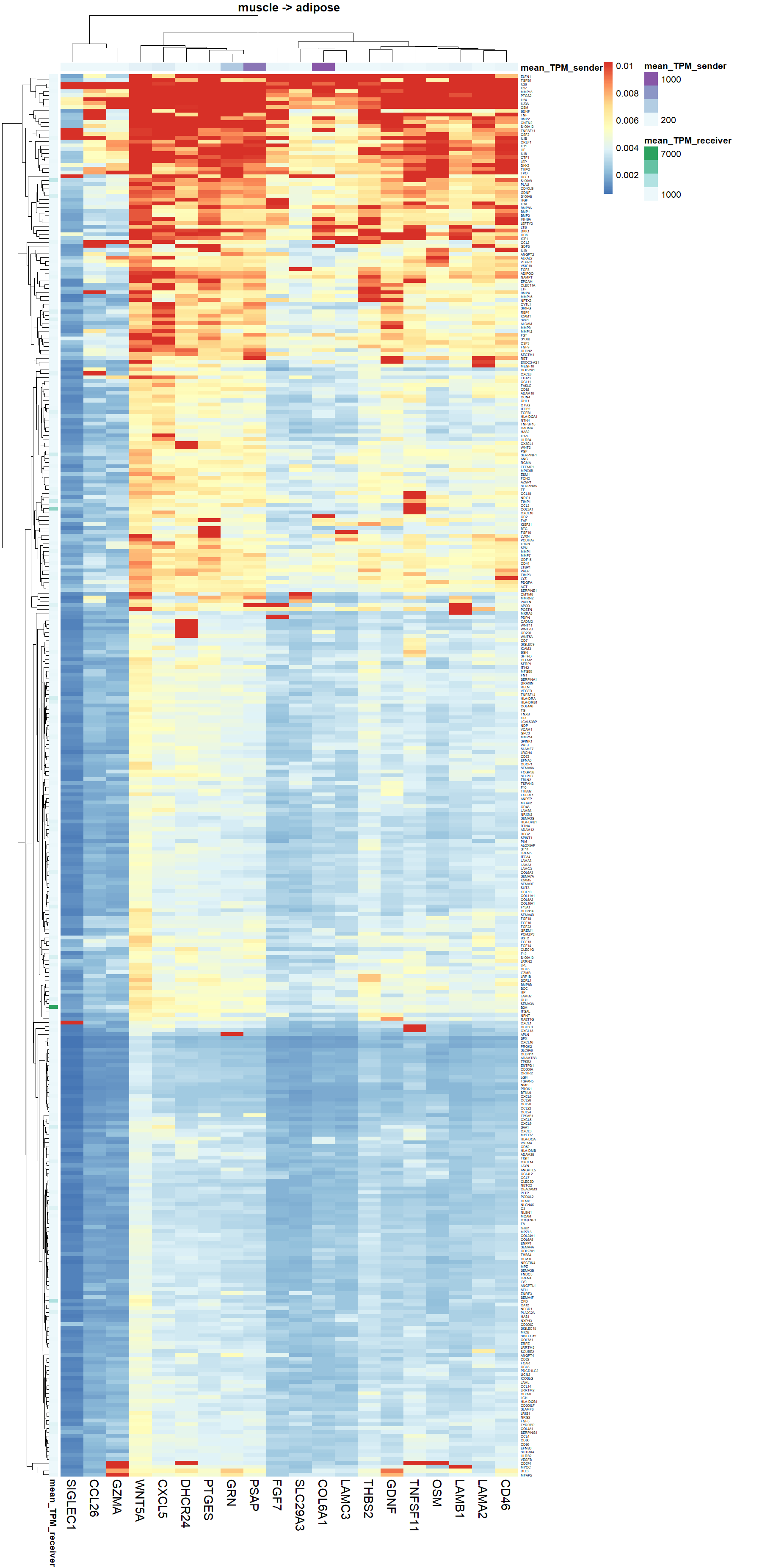


**Figure S9. Muscle to adipose paracrine signaling.**

Heatmap of ligand–receptor expression pairs highlighting muscle-derived signals targeting adipose receptors.
